# Mathematical model analysis of effective intervention strategies on transmission dynamics of hepatitis B virus

**DOI:** 10.1038/s41598-023-35815-z

**Published:** 2023-05-30

**Authors:** Firaol Asfaw Wodajo, Dawit Melesse Gebru, Haileyesus Tessema Alemneh

**Affiliations:** 1 Department of Mathematics, Debre Berhan University, Debre Berhan, Ethiopia; 2 Department of Mathematics, Bahirdar University, Bahirdar, Ethiopia; 3 Department of Mathematics, University of Gondar, Gondar, Ethiopia

**Keywords:** Computational biology and bioinformatics, Diseases

## Abstract

Hepatitis B is one of the world’s most common and severe infectious diseases. Worldwide, over 350 million people are currently estimated to be persistent carriers of the hepatitis B virus (HBV), with the death of 1 million people from the chronic stage of HBV infection. In this work, developed a nonlinear mathematical model for the transmission dynamics of HBV. We constructed the mathematical model by considering vaccination, treatment, migration, and screening effects. We calculated both disease-free and endemic equilibrium points for our model. Using the next-generation matrix, an effective reproduction number for the model is calculated. We also proved the asymptotic stability of both local and global asymptotically stability of disease-free and endemic equilibrium points. By calculating the sensitivity indices, the most sensitive parameters that are most likely to affect the disease’s endemicity are identified. From the findings of this work, we recommend vaccination of the entire population and screening all the exposed and migrants. Additionally, early treatment of both the exposed class after screening and the chronically infected class is vital to decreasing the transmission of HBV in the community.

## Introduction

There are various deadly infectious diseases that affect the lives of people around the world, including hepatitis B^1^. Hepatitis B is an infection caused by the hepatitis B virus (HBV) that damages the liver and can cause both acute and chronic infections. Although many people with hepatitis B do not experience symptoms in the early stages of infection, some develop sudden symptoms such as vomiting, yellowing of the skin, fatigue, black urine, and abdominal pain^2,3^. Approximately one-third of the world's population is infected at some point in their lifetime, and 240–350 million people suffer from chronic infections^2^. Compared to HIV, which has captivated both the public and health planners and spurred a global effort, HBV and HCV, which are essentially equally transmissible, are 100 times more infectious than hers and 10 times more infectious, respectively^4^. In countries where HBV is moderately to highly endemic, the most common routes of transmission are mother-to-child transmission, percutaneous or other contact with contaminated blood or body fluids, or sexual intercourse^4,5^.

In 2015, 900,000 deaths from hepatitis B virus (HBV) infection, mainly from the development of cirrhosis and hepatocellular carcinoma (HCC), were recorded^6^. Birth vaccination with hepatitis B is the cornerstone for preventing perinatal and horizontal HBV transmission. Health systems need to improve viral hepatitis prevention and treatment programs^7,8^. Antiviral therapies such as lamivudine, telbivudine, and tenofovir are being investigated as potential interventions to reduce perinatal HBV infection in pregnant women with high HBV DNA levels.

HBV screening is important because HBV is a highly contagious disease with serious public health consequences, and vaccination could prevent her from getting HBV infection and its complications^9^. The goals of HBV screening are to identify chronically infected individuals who may benefit from treatment and/or education about necessary lifestyle changes to prevent transmission, HBV screening, and the HBV vaccine. To identify the close contacts of an infected person for vaccination. care in cases of CHB and chronically infected persons for monitoring disease activity and HCC surveillance^10^. Targeted screening of risk groups, including migrants, may help improve case detection. The cost-effectiveness of viral hepatitis screening in immigrants from countries with moderate or high prevalence has been evaluated, reinforcing the need for screening as a form of secondary prevention^11^.

Post-exposure prophylaxis (PEP) is key to preventing infection after HBV exposure. PEP is used to reduce the risk of infection with the hepatitis B virus. PEP with HBV vaccination alone or combined with HBV vaccination and passive immunization with HBV immunoglobulin (HBIG) has been shown to be highly effective in preventing HBV transmission after exposure to infected blood or body fluids. It has been. B. through contaminated sharp injury, sexual contact, or perinatal exposure^12^. The movement of people within and between countries is one of many factors contributing to changes in the epidemiology of viral hepatitis^13^. In today's world of rapid global migration, any disease can spread faster than ever before. Migrants have traditionally played an important role in the spread of epidemics. According to^8^, approximately 40,000 chronic hepatitis B patients entered the United States each year between 1994 and 2003. According to a recent review by the European Center for Disease Prevention and Control, the population prevalence of CHB varies widely across European countries, ranging from 0.1% in Ireland and the Netherlands to 0.7% in eastern Turkey^14^. The review also found that the estimated prevalence of CHB infection was higher in immigrant groups than in the general population in all countries for which data were available^15^. The Institute of Medicine (IOM) of the National Academy recently proposed a national strategy for the prevention and control of hepatitis B and C, highlighting the importance of HBV screening and vaccination^16^.

In recent years, mathematical and statistical models of infectious diseases have provided useful insights into the dynamics and control of infectious disease transmission^17^. A mathematical model of the dynamics of disease transmission is needed to provide better insight into disease behavior, optimize the use of limited resources, and recommend infectious disease control approaches^18^. A mathematical model of the infectivity dynamics of each disease is essential to gain better insight into disease behavior. It can recommend measures to optimize the use of limited resources and combat infections. Mathematical and statistical models of infectious diseases have historically provided useful insights into the dynamics of transmission and control of these diseases. Anderson et al.^19^ used a simple deterministic compartmentalization mathematical model to show the influence of carriers on HBV transmission. Medley et al.^4^ developed a model showing that the prevalence of infection is largely driven by a feedback loop involving infection rate, average age at infection, and age-related risk of infection after infection. Zou et al.^20^ used a discrete dynamic model to assess the independent impact of neonatal vaccination on reducing HBV incidence in China. To assess hepatitis B epidemiology in New Zealand, McMahon et al.^21^ classified the total population into five age groups. Given the importance of age in HBV infection, a PDE model was constructed to characterize infection, assess the long-term efficacy of vaccination regimens, and study transmissibility dynamics. Sisodiya et al.^22^ proposed and analyzed a non-autonomous mathematical model for water-borne disease. The suggested model comprised three temperature-dependent factors that were related to the development and death rates of pathogens in aquatic environments as well as the rate at which those pathogens transmitted disease to humans. To explain the extinction or persistence of the sickness, they developed a threshold condition in terms of RC(t). They used numerical simulation to demonstrate our theoretical findings and forecast how well the control measures would work.

Misra and Sisodiya^23^ proposed a mathematical model to study the transmission and control of COVID-19. It includes disease-related parameters such as contact rates, death rates, immigration rates, transition rates, and control measures such as social distancing practices, isolation, and quarantine rates. Social distancing and constant immigration of asymptomatic infected persons can prevent the spread of COVID-19, while vaccination and testing rates can have synergistic effects on minimizing COVID-19 cases. Sisodiya et al.^24^ proposed a delayed SEIRB epidemic model with impulsive vaccination and disinfection. To combat cholera, they researched cleanliness and the pulse vaccination approach. Using the impulsive dynamical system represented by the stroboscopic map, they have demonstrated the existence of an infection-free periodic solution. They were able to derive a necessary condition for the epidemic's persistence with pulse vaccination from the model's analysis. Sisodiya et al.^25^ proposed a mathematical model to study the spread of pathogen-induced cholera disease and its control by vaccination. In the model, they presuppose that immunization against cholera has a transient impact and that recipients of a subpar vaccination dosage are not protected. To demonstrate the relative significance of system factors on disease transmission and prevalence, they conducted sensitivity analyses on those parameters. Khan et al.^26^ developed A stochastic *SACR* epidemic model for HBV transmission. They developed a stochastic epidemic model by considering the effect of environmental fluctuation on hepatitis B dynamics and distributing the transmission rate by white noise. From their analysis, they proved that the intensity of the noise has a significant influence on the transmission of the disease, which is verified using the Itô-Taylor stochastic scheme.

Khan et al.^27^ created HBV epidemic model with convex incidence rate: modeling and sensitivity analysis." The authors used numerical simulations to demonstrate the viability of a control approach and its efficacy in eradicating Hepatitis B and they came to the conclusion that, with the long-term application of such a control plan, Hepatitis B might be completely eradicated from society.

Yavuz et al.^28^ proposed A New Modeling of Fractional-Order and Sensitivity Analysis for Hepatitis-B Disease with Real Data. The findings show that the dynamical behavior of the developed model for hepatitis B is significantly influenced by the order of the fractional derivative.

Liu et al.^29^ proposed Numerical dynamics and fractional modeling of HBV model with non-singular and non-local kernels. They developed an Atangana-Baleanu derivative (AB derivative) fractional hepatitis B viral model incorporating vaccination effects. According to their findings, receiving vaccinations is an efficient method of curing Hepatitis B in the general population, making specialized vaccination procedures and treatments for infectious diseases extremely important.

None of the above-reviewed literature considered screening migration in their work. By making model on^19^ as a benchmark we developed our model by adding treatment at two stages and screening at two stages. This makes this manuscript different from the previous works on the transmission dynamics of HBV. Based on these facts, we make assumptions about the screening of exposed individuals and migrated classes, which play an important role in reducing HBV disease transmission, and population migration, which currently contributes significantly to the global transmission of infectious diseases. has been incorporated. We also considered treatment both at the chronic stage and after the screening, leading to the recovery of individuals in our model. This manuscript intends to answer the following questions:

1. What are the most sensitive parameters contributing a lot to the change in the effective reproduction number of HBV?
2. What are the contributions that the combined effects of screening, vaccination, and treatment make to the decrement of transmission of HBV?
3. What recommendations should be forwarded to the stakeholders to change the endemicity of HBV to non-endemic?

## Mathematical model and analysis

Based on the nature of HBV disease, we divided the entire population into eight compartments namely:$S(t)$—Susceptible, $V(t)$—Vaccinated, $E(t)$—Exposed, $Q(t)$—Screened, $M(t)$—Migrated, $A(t)$—Acutely infected, $C(t)$—Chronically infected, $T(t)$—Treated and $R(t)$—Recovered population. We considered both horizontal and vertical transmission mode of HBV virus.

The birth flux is recruited into the susceptible compartment by the rate $b\left(1-\omega \right)\left(1-\gamma C\right)$ and decreases by the interaction and contact with acutely and chronically infected population with force of infection $\lambda =\rho \left(A+\pi C\right)$. $b\left(1-\omega \right)\left(1-\gamma C\right)$ shows that the newborns are unimmunized and become susceptible again where *b* is birth rate. $b\omega$ measures the successful immunization of newborn babies, $\gamma$ is the rate by which the unimmunized children born to carrier mothers. $\theta$ represents the vaccination of susceptible individuals. Exposed population increases with force of infection. Exposed population goes to screened compartment by $\zeta \sigma \beta$. The remaining joins acutely and chronically infected population with rates of $\left(1-\sigma \right)\beta$ and $\left(1-\zeta \right)\sigma \beta$ respectively. If screened population do not get early treatment, they join acutely infected compartment by rate of $\left(1-\alpha \right)\phi$, those who get treatment early after will recover by the rate $\alpha \phi$. Acutely infected population recovers by natural immunity by the rate $(1-\nu ){\beta }_{1}$ where the remaining part joins chronically infected compartment by the rate $\nu {\beta }_{1}$. Chronically infected population goes for treatment by the rate $\eta$. $\pi$ represents the level of carrier infectiousness to acute infection. We assume that the population of newborn carriers born to carriers’ mothers is less than the sum of the disease induced death rate $d,$ and the population moving from chronic infective class to treated class,$\eta$. In this case we have $b\gamma \left(1-\omega \right)<\mu +d+\eta$ . Otherwise, carriers would keep increasing rapidly as long as there is infection; that is $\frac{dC}{dt}>0 for C\ne 0$ or $A\ne 0$ and $t\ne 0$. Chronically infective population who got successful treatment recovers by the rate $\delta .$ We also included the fact, the immunity after recovery is lifetime and vaccination might wane after some time. Since migration plays a great role in transmission of infectious disease in the host country, migrated class move to screened class by the rate of $k\varepsilon {\delta }_{1}$, join exposed class by the rate $\left(1-k\right){\delta }_{1}$ and acutely infected class by the rate $\left(1-\varepsilon \right)k{\delta }_{1}$. Each compartment has natural death rate $\mu .$

**Figure 1:**
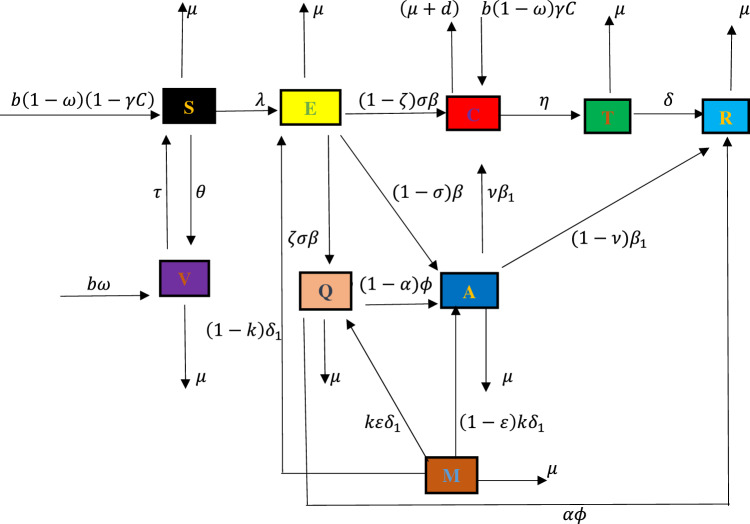
Flow chart of HBV model.

Considering the above assumptions and the compartmental flow diagram in Fig. 1 above, the model is governed by the following system of nonlinear ordinary differential equations.

1 $$\begin{aligned} \frac{dS}{dt} &=b\left(1-\omega \right)\left(1-\gamma C\right)+\tau V-\left(\lambda +\theta +\mu \right)S \\ \frac{dV}{dt} & =b\omega +\theta S-\left(\tau +\mu \right)V \\ \frac{dE}{dt} & =\lambda S+\left(1-k\right){\delta }_{1}M-\left(\beta +\mu \right)E \\ \frac{dQ}{dt} & =\zeta \sigma \beta E+k\varepsilon {\delta }_{1}M-\left(\phi +\mu \right)Q \\ \frac{dM}{dt} & =-\left({\delta }_{1}+\mu \right)M \\ \frac{dA}{dt} & =\left(1-\sigma \right)\beta E+\left(1-\varepsilon \right)k{\delta }_{1}M+\left(1-\alpha \right)\phi Q-\left({\beta }_{1}+\mu \right)A \\ \frac{dC}{dt} & =b\left(1-\omega \right)\gamma C+\left(1-\zeta \right)\sigma \beta E+\nu {\beta }_{1}A-\left(\mu +d+\eta \right)C \\ \frac{dT}{dt} & =\eta C-\left(\delta +\mu \right)T \\ \frac{dR}{dt} &=\left(1-\nu \right){\beta }_{1}A+\delta T+\alpha \phi Q-\mu R \end{aligned}$$

with initial conditions,

$$S\left(0\right)\ge 0,V\left(0\right)\ge 0,E\ge 0,Q\left(0\right)\ge 0,M\left(0\right)\ge 0,A\left(0\right)\ge 0,C\left(0\right)\ge 0,T\left(0\right)\ge 0,$$ and $$R(0)\ge 0$$.

We use the parametric values given in Table 1 below for sensitivity index and numerical simulation.

**Table 1:** Parametric description and their values.

Parameter	Description	Values	Units	Sources
$b$	Birth rate	0.0121*${N}_{0}$	$Size*{Time }^{-1}$	^12^
$\omega$	Proportion of births with successful vaccination	0.52	${Time }^{-1}$	^20^
$\gamma$	Proportion of perinatally infected (carrier mothers)	0.11	${Time }^{-1}$	^30^
$\rho$	Contact rate	0.33	$Size*{Time }^{-1}$	^20^
ζ	Proportion by which exposed individuals goes for treatment after screening	0.115	${Time }^{-1}$	^2^
$\sigma$	Proportion of exposed individuals that progress to chronic Stage	0.08	${Time }^{-1}$	^31^
$\beta$	Progression rate from exposed class to acute class	0.0164	${Time }^{-1}$	^30^
$\alpha$	Proportion of screened individuals moving recovery	0.27	${Time }^{-1}$	Assumed
$\phi$	Progression rate from screened class to recovered class	0.31	${Time }^{-1}$	Assumed
$\mu$	Natural death rate	0.00693	${Time }^{-1}$	^32^
${\beta }_{1}$	Progression rate from acute to chronic stage	0.0109	${Time }^{-1}$	^30^
$\tau$	Rate of waning of vaccination efficacy	0.1	${Time }^{-1}$	^33^
$\theta$	The rate by which susceptible population get vaccinated	0.5	${Time }^{-1}$	^30^
ν	Average probability an individual fails to clear an acute infection and develops to carrier state	0.885	${Time }^{-1}$	^5^
$k$	Screening rate of migrated class	0.23	${Time }^{-1}$	Assumed
${\delta }_{1}$	Progression rate from migrants to acute stage	0.37	${Time }^{-1}$	^13^
$\varepsilon$	Proportion by which migrated individuals get vaccinated	0.56	${Time }^{-1}$	^13^
$d$	HBV related death rate	0.002	${Time }^{-1}$	^12^
$\eta$	Rate by which chronically infected goes for treatment	0.28	${Time }^{-1}$	^30^
$\delta$	Recovery rate of treated individuals	0.32	${Time }^{-1}$	^34^
$\pi$	Level of infectiousness of chronic infective	0.5	${Time }^{-1}$	^30^

### Positivity and boundedness of solutions

#### Positivity

##### **Theorem 1**

*Let* $\Omega =\left\{\left(S,V,E,Q,M,A,C,T,R\right)\in {{R}_{+}}^{9}:N\le \frac{b}{\mu }: {S}_{0}\ge 0,{V}_{0}\ge 0,{E}_{0}\ge 0,{Q}_{0}\ge 0,{M}_{0}\ge 0,{A}_{0}{\ge 0,C}_{0}{\ge 0,T}_{0}\ge 0,{R}_{0}\ge 0\right\}$ *then, the solutions* $S\left(t\right),V\left(t\right),E\left(t\right),Q\left(t\right),M\left(t\right),A\left(t\right),C\left(t\right),T(t)$ *and* $R(t)$ *of the model will be remaining positive for all time* $t>0.$

##### *Proof*

Here we will discuss positivity of the population with initial conditions ${S}_{0}\ge 0,{V}_{0}\ge 0,{E}_{0}\ge 0,{Q}_{0}\ge 0,{M}_{0}\ge 0,{A}_{0}{\ge 0,C}_{0}{\ge 0,T}_{0}\ge 0,{R}_{0}\ge 0.$

From first equation of (1), $\frac{dS}{dt}=b\left(1-\omega \right)\left(1-\gamma C\right)+\tau V-\left(\lambda +\theta +\mu \right)S=0$, we get:

$$\Rightarrow \frac{dS}{dt}+\left(\lambda +\theta +\mu \right)S=b\left(1-\omega \right)\left(1-\gamma C\right)+\tau V$$

Then, we calculate for Integrating factor $,I.F={e}^{\int \left(\lambda +\theta +\mu \right)dt}$

Multiplying the equation $\frac{dS}{dt}+\left(\lambda +\theta +\mu \right)S=b\left(1-\omega \right)\left(1-\gamma C\right)+\tau V$ by integrating factor, we get:

$${e}^{\int \left(\lambda +\theta +\mu \right)dt}\frac{dS}{dt}+{e}^{\int \left(\lambda +\theta +\mu \right)dt}\left(\lambda +\theta +\mu \right)S={e}^{\int \left(\lambda +\theta +\mu \right)dt}\left(b\left(1-\omega \right)\left(1-\gamma C\right)+\tau V\right)$$

Integrating both sides $\frac{d}{dt}\left(S\left(t\right){.e}^{{\int }_{0}^{\psi }\left(\lambda +\theta +\mu \right)dt}\right)={e}^{{\int }_{0}^{\psi }\left(\lambda +\theta +\mu \right)dt}\left(b\left(1-\omega \right)\left(1-\gamma C\right)+\tau V\right)$

$${\left.\left(S(t){.e}^{{\int }_{0}^{\psi }\left(\lambda +\theta +\mu \right)dt}\right)\right|}_{0}^{\psi }={\int }_{0}^{\psi }{e}^{{\int }_{0}^{\psi }\left(\lambda +\theta +\mu \right)dt}\left(b\left(1-\omega \right)\left(1-\gamma C\right)+\tau V\right)dt$$

$${\left.\left(S(t){.e}^{\left(\theta +\mu \right)t+{\int }_{0}^{\psi }\lambda \left(u\right)du}\right)\right|}_{0}^{\psi }={\int }_{0}^{\psi }{e}^{{\int }_{0}^{\psi }\left(\lambda +\theta +\mu \right)dt}\left(b\left(1-\omega \right)\left(1-\gamma C\right)+\tau V\right)dt$$

$$S(\psi ){.e}^{\left(\theta +\mu \right)t+{\int }_{0}^{\psi }\lambda \left(u\right)du}-S\left(0\right)={\int }_{0}^{\psi }{e}^{{\int }_{0}^{\psi }\left(\lambda +\theta +\mu \right)dt}\left(b\left(1-\omega \right)\left(1-\gamma C\right)+\tau V\right)dt$$

$$S\left(\psi \right)={K}_{1}S\left(0\right)+{K}_{1}{\int }_{0}^{\psi }{e}^{{\int }_{0}^{\psi }\left(\lambda +\theta +\mu \right)dt}.\left(b\left(1-\omega \right)\left(1-\gamma C\right)+\tau V\right)dt>0.$$

Since ${K}_{1}={e}^{-\left(\mu +\theta \right)t}+{\int }_{0}^{\psi }\lambda \left(w\right)dw>0,S\left(0\right)\ge 0$ and from the definition of $\psi$ and $V\left(t\right)\ge 0,b\left(1-\omega \right)\left(1-\gamma C\right)>0,S\left(\psi \right)>0.$ Thus, based on the definition of $\tau$ above, $\tau$ is not finite which means $\tau =+\infty$ $S(t)\ge 0.$ The same is true for $V$(t)$\ge 0$, $E$(t)$\ge 0$, $Q$(t)$\ge 0$, $M$(t)$\ge 0$, $A$(t) $\ge 0$, $C$(t) $\ge 0$, $T$(t)$\ge 0$, $R$(t)$\ge 0.$

#### Boundedness

##### **Theorem 2**

*All feasible solutions of the model* (1) *are uniformly bounded in a proper subset* $\Omega =\left\{\left(S,V,E,Q,M,A,C,T,R\right)\in {{R}_{+}}^{9}:N\le \frac{b}{\mu }\right\}$.

##### *Proof*

To show the boundedness of the solution, we have to show a lower bound and upper bound. But, initially,$N\left(0\right)={N}_{0}>0,S\left(0\right)={S}_{0}\ge 0,V\left(0\right)={V}_{0}\ge 0, E\left(0\right)={E}_{0}\ge 0,Q\left(0\right)={Q}_{0}\ge 0,M\left(0\right)={M}_{0}\ge 0 ,A\left(0\right)={A}_{0}\ge 0, C\left(0\right)={C}_{0}\ge 0,T\left(0\right)={T}_{0}\ge 0,R\left(0\right)={R}_{0}\ge 0$.

Here, we consider boundedness of our HBV model under initial conditions using $N=S+V+E+Q+M+A+C+T+R$, and we have:

$$\frac{dN}{dt}=\frac{dS}{dt}+\frac{dV}{dt}+\frac{dE}{dt}+\frac{dQ}{dt}+\frac{dM}{dt}+\frac{dA}{dt}+\frac{dC}{dt}+\frac{dT}{dt}+\frac{dR}{dt}$$

and adding this system of differential equations and simplification yields:

$$\Rightarrow \frac{dN}{dt}=b - \mu N - dC$$

$\Rightarrow \frac{dN}{dt}\le b-\mu N$, Integrating both sides, applying the initial condition $N(0)={N}_{0}$ and solving gives us: $N\le \frac{b}{\mu }.$

This implies that $0\le N\le \frac{b}{\mu }.$ Thus, feasible solution set of the system equation of the model enters and remains in the region $\Omega =\left\{\left(S,V,E,Q,M,A,C,T,R\right)\in {{R}_{+}}^{9}:N\le \frac{b}{\mu }\right\}$.

### Disease-free equilibrium point (DFEP) $\left({D}^{0}\right)$

In order to calculate Disease free equilibrium point; point at which the epidemic is eradicated from the population. we use $E=Q=A=C=Q =0$, in the system of differential Eq. (1).

From (1) above, Then, DFEP of our model becomes:

$${D}^{0}=\left({S}^{0},{{V}^{0},E}^{0}{,M}^{0},{{Q}^{0},A}^{0}{,C}^{0}{,T}^{0},{R}^{0}\right)=\left({S}^{0},{V}^{0},\mathrm{0,0},\mathrm{0,0},\mathrm{0,0},0\right)$$ where $${S}^{0}=\frac{b\left(\tau -\mu \left(1-\omega \right)\right)}{\mu \left(\theta +\tau +\mu \right)}$$ and

$${V}^{0}=\frac{b\left(\left(1-\omega \right)\mu \left(\theta +\tau +\mu \right)+\left(\theta +\omega \right)\left(1-\mu \left(1-\omega \right)\right)\right)}{\tau \mu \left(\theta +\tau +\mu \right)}.$$

### Effective reproduction number (${{\varvec{R}}}_{{\varvec{E}}{\varvec{f}}{\varvec{f}}}$)

The effective reproduction number (${R}_{Eff}$) is used to measure the transmission potential of a disease. It is reproduction number of HBV with screening, vaccination and treatment interventions used in the model. It is the average number of secondary infections produced by a typical case of an infection in a population where everyone is susceptible^35^. To calculate effective reproduction number of our HBV model, we used the next-generation matrix method so that it is the spectral radius of the next-generation matrix, $F{V}^{-1}=\left[\partial {\mathcal{F}}_{i}\left(\frac{{E}^{1}}{\partial {x}_{j}}\right)\right]{\left[\partial {v}_{i}\left(\frac{{E}^{1}}{\partial {x}_{j}}\right)\right]}^{-1}.$ The effective reproduction number, ${R}_{Eff}$ of our HBV model is obtained by rearranging the differential equation of the dynamical system (1) above in terms of $\frac{d{X}_{i}}{dt}={\mathcal{F}}_{i}-{v}_{i}={\mathcal{F}}_{i}-\left({v}_{i}^{-}-{v}_{i}^{+}\right)$.

$$F=\left[\begin{array}{cccc}0& 0& {Y}_{1}& \theta {Y}_{1}\\ 0& 0& 0& 0\\ 0& 0& 0& 0\\ 0& 0& 0& 0\end{array}\right];$$

$$V=\left[\begin{array}{cccc}\left(\beta +\mu \right)& 0& 0& 0\\ -\zeta \sigma \beta & \left(\phi +\mu \right)& 0& 0\\ -\left(1-\sigma \right)\beta & -\left(1-\alpha \right)\phi & \left({\beta }_{1}+\mu \right)& 0\\ -\left(1-\zeta \right)\sigma \beta & 0& -\nu {\beta }_{1}& {Y}_{2}\end{array}\right];$$

where $${Y}_{1}=\rho {S}^{0},{Y}_{2}=\left(\mu +d+\eta -b\gamma \left(1-\omega \right)\right)$$

$$V^{ - 1} = \left[ {\begin{array}{*{20}c} {X_{1} } & 0 & 0 & 0 \\ {X_{2} } & {\frac{1}{\mu + \varphi }} & 0 & 0 \\ {X_{3} } & {X_{5} } & {\frac{1}{{\mu + \beta_{1} }}} & 0 \\ {X_{4} } & {X_{6} } & {X_{7} } & {\frac{1}{{Y_{2} }}} \\ \end{array} } \right];$$

where $${X}_{1}=\frac{1}{\beta +\mu }, {X}_{2}=\frac{\beta \zeta \mu \sigma {Y}_{2}+\beta \zeta \sigma {Y}_{2}{\beta }_{1}}{\left(\beta +\mu \right)\left(\mu +\phi \right){Y}_{2}\left(\mu +{\beta }_{1}\right)},{X}_{3}=\frac{\beta \mu {Y}_{2}-\beta \mu \sigma {Y}_{2}+\beta \phi {Y}_{2}-\beta \sigma \phi {Y}_{2}+\left(1-\alpha \right)\beta \zeta \sigma \phi {Y}_{2}}{\left(\beta +\mu \right)\left(\mu +\phi \right){Y}_{2}\left(\mu +{\beta }_{1}\right)},$$ $${X}_{4}=\frac{\nu \beta \left(\left(1-\sigma \right)\left(\left(\mu +\phi \right)+\zeta \sigma \phi \right)\right){\beta }_{1}+(1-\zeta )\sigma (\mu +\phi )(\mu +{\beta }_{1})}{(\beta +\mu )(\mu +\phi ){Y}_{2}(\mu +{\beta }_{1})},$$ $${X}_{5}=\frac{\left(1-\alpha \right)\phi {Y}_{2}\left(\beta +\mu \right)}{(\beta +\mu )(\mu +\phi ){Y}_{2}(\mu +{\beta }_{1})},{X}_{6}=\frac{\left(1-\alpha \right){\nu \phi \beta }_{1}\left(\beta +\mu \right)}{(\beta +\mu )(\mu +\phi ){Y}_{2}(\mu +{\beta }_{1})},{X}_{7}=\frac{\nu {\beta }_{1}}{{Y}_{2}(\mu +{\beta }_{1})}.$$

$$F{V}^{-1}=\left[\begin{array}{cccc}{X}_{3}{Y}_{1}+\theta {X}_{4}{Y}_{1}& {X}_{5}{Y}_{1}+\theta {X}_{6}{Y}_{1}& \theta {X}_{7}{Y}_{1}+\frac{{Y}_{1}}{\mu +{\beta }_{1}}& \frac{\theta {Y}_{1}}{{Y}_{2}}\\ 0& 0& 0& 0\\ 0& 0& 0& 0\\ 0& 0& 0& 0\end{array}\right]$$

Determinant of the matrix is given by:

$$\left|\begin{array}{ccccc}0-\lambda & 0& 0& {X}_{1}{Y}_{1}& \theta {X}_{1}{Y}_{1}\\ 0& 0-\lambda & 0& {X}_{2}{Y}_{1}& \theta {X}_{2}{Y}_{1}\\ 0& 0& 0-\lambda & 0& 0\\ 0& 0& 0& {X}_{3}{Y}_{1}-\lambda & \theta {X}_{3}{Y}_{1}\\ 0& 0& 0& {X}_{4}{Y}_{1}& \theta {X}_{4}{Y}_{1}-\lambda \end{array}\right|=0.$$

Eigenvalues of the determinant are: $\left\{{\lambda }_{1,}{\lambda }_{2,}{\lambda }_{3,}{\lambda }_{4}\right\}=\{\mathrm{0,0},0,({X}_{3}+\theta {X}_{4}){Y}_{1}\}$

Then, the spectral radius is: $Max\left\{{\lambda }_{1,}{\lambda }_{2,}{\lambda }_{3,}{\lambda }_{4}\right\}=Max\{$ $\{\mathrm{0,0},0,({X}_{3}+\theta {X}_{4}){Y}_{1}\}=\left({X}_{3}+\theta {X}_{4}\right){Y}_{1}$

$${R}_{Eff}=\frac{b\rho \beta \left(\tau -\mu \left(1-\omega \right)\right)\left[\left(\mu \left(1-\sigma \right)+\phi \left(1-\sigma \left(1+\left(1-\alpha \right)\zeta \right)\right)\right)\left(\mu +d+\eta -b\gamma \left(1-\omega \right)+\theta \nu {\beta }_{1}\right)+\sigma (1-\zeta )(\mu +\phi )(\mu +{\beta }_{1})\right]}{\mu \left(\theta +\tau +\mu \right)(\beta +\mu )(\mu +\phi )(\mu +{\beta }_{1})\left(\mu +d+\eta -b\gamma \left(1-\omega \right)\right)}.$$

### Local stability analysis of DFEP (${D}^{0}$)

#### **Theorem 3**

*If* ${R}_{Eff}<1, {D}^{0}$ *is locally asymptotically stable; if* ${R}_{Eff}>1$ *it is unstable*.

#### *Proof*

To show local stability of Disease-free equilibrium point of our model, we did as follow. The Jacobean matrix is given by:

$$J\left({D}^{0}\right)=\left[\begin{array}{ccccccccc}-(\theta +\mu )& \tau & 0& 0& 0& -{R}_{1}& -\pi {R}_{1}-b(1-\omega )\gamma & 0& 0\\ \theta & -(\tau +\mu )& 0& 0& 0& 0& 0& 0& 0\\ 0& 0& -(\beta +\mu )& 0& \left(1-k\right){\delta }_{1}& {R}_{1}& \pi {R}_{1}& 0& 0\\ 0& 0& \zeta \sigma \beta & -(\phi +\mu )& k\varepsilon {\delta }_{1}& 0& 0& 0& 0\\ 0& 0& 0& 0& -({\delta }_{1}+\mu )& 0& 0& 0& 0\\ 0& 0& (1-\sigma )\beta & (1-\alpha )\phi & (1-\varepsilon )k{\delta }_{1}& -({\beta }_{1}+\mu )& 0& 0& 0\\ 0& 0& (1-\zeta )\sigma \beta & 0& 0& \nu {\beta }_{1}& -{Z}_{2}& 0& 0\\ 0& 0& 0& 0& 0& 0& \eta & -(\delta +\mu )& 0\\ 0& 0& 0& \mathrm{\alpha }\phi & 0& (1-\nu ){\beta }_{1}& 0& \delta & -\mu \end{array}\right]$$

Let $${R}_{1}=\rho {S}^{0},{Z}_{2}=\left(\eta +d+\mu \right)-b\left(1-\omega \right)\gamma$$

Determinant of the Jacobean matrix is given by:

$$\left|\begin{array}{ccccccccc}-(\theta +\mu )-\lambda & \tau & 0& 0& 0& -{R}_{1}& -\pi {R}_{1}-b(1-\omega )\gamma & 0& 0\\ \theta & -(\tau +\mu )-\lambda & 0& 0& 0& 0& 0& 0& 0\\ 0& 0& -(\beta +\mu )-\lambda & 0& \left(1-k\right){\delta }_{1}& {R}_{1}& \pi {R}_{1}& 0& 0\\ 0& 0& \zeta \sigma \beta & -(\phi +\mu )-\lambda & k\varepsilon {\delta }_{1}& 0& 0& 0& 0\\ 0& 0& 0& 0& -({\delta }_{1}+\mu )-\lambda & 0& 0& 0& 0\\ 0& 0& (1-\sigma )\beta & (1-\alpha )\phi & (1-\varepsilon )k{\delta }_{1}& -({\beta }_{1}+\mu )-\lambda & 0& 0& 0\\ 0& 0& (1-\zeta )\sigma \beta & 0& 0& \nu {\beta }_{1}& -{Z}_{2}-\lambda & 0& 0\\ 0& 0& 0& 0& 0& 0& \eta & -(\delta +\mu )-\lambda & 0\\ 0& 0& 0& \mathrm{\alpha }\phi & 0& (1-\nu ){\beta }_{1}& 0& \delta & -\mu -\lambda \end{array}\right|=0$$

Then, the characteristic equation is:

Let $$P\left(\lambda \right)={l}_{0}{\lambda }^{5}+{l}_{1}{\lambda }^{4}+{l}_{2}{\lambda }^{3}+{l}_{3}{\lambda }^{2}+{l}_{4}\lambda +{l}_{5}=0$$

Where: $${l}_{0}=1$$

$${l}_{1}=\left(\beta +3\mu +\phi +{Z}_{2}+{\beta }_{1}+k\varepsilon {\delta }_{1}\right)$$

$${l}_{2}=\left(\left(2\beta +3\mu +2\phi \right)\mu +\beta \phi -\left(1-\sigma +\pi \sigma -\pi \zeta \sigma \right)\beta {R}_{1}+\left(3\mu +\phi \right){Z}_{2}+\left(\beta +2\mu +\phi +{Z}_{2}\right){\beta }_{1}\right),$$

$${l}_{3}=\left(\beta {\mu }^{2}+{\mu }^{3}+\beta \mu \phi +{\mu }^{2}\phi -\beta \mu {R}_{1}+\beta \mu \sigma {R}_{1}-2\pi \beta \mu \sigma {R}_{1}+2\pi \beta \zeta \mu \sigma {R}_{1}-\beta \phi {R}_{1}+\beta \sigma \phi {R}_{1}-\pi \beta \sigma \phi \left(1-k\right){\delta }_{1}{R}_{1}+\pi \beta \zeta \sigma \phi {R}_{1}-\alpha \beta \zeta \sigma \phi {\left(1-k\right){\delta }_{1}R}_{1}+2\beta \mu {Z}_{2}+2\mu \phi {Z}_{2}++\beta \mu {\beta }_{1}+{\mu }^{2}{\beta }_{1}+\beta \phi {\beta }_{1}+\mu \phi {\beta }_{1}-\pi \beta \nu (1-\varepsilon )k{\delta }_{1}{R}_{1}{\beta }_{1}-\pi \beta \sigma {R}_{1}{\beta }_{1}+\pi \beta \zeta \sigma {R}_{1}{\beta }_{1}-\beta {R}_{1}{Z}_{2}\right),$$

$${l}_{4}=\left(\beta \sigma {Z}_{1}{Z}_{2}+\beta \phi {Z}_{2}+\pi \beta \nu \sigma (1-\varepsilon )k{\delta }_{1}{R}_{1}{\beta }_{1}+\beta {Z}_{2}{\beta }_{1}+2\mu {Z}_{2}{\beta }_{1}+\phi {Z}_{2}{\beta }_{1}+3{\mu }^{2}{Z}_{2}-\pi \beta {\mu }^{2}\sigma {R}_{1}+\pi \beta \zeta {\mu }^{2}\sigma {R}_{1}-\pi \beta \mu \sigma \phi {R}_{1}+\pi \beta \zeta \mu \sigma \phi {R}_{1}+\beta {\mu }^{2}{Z}_{2}+{\mu }^{3}{Z}_{2}+\beta \mu \phi {Z}_{2}+{\mu }^{2}\phi {Z}_{2}-\beta \mu {R}_{1}{Z}_{2}++\beta \mu \sigma {(1-\varepsilon )k{\delta }_{1}R}_{1}{Z}_{2}+\beta \phi {R}_{1}{Z}_{2}+\beta \sigma \phi {R}_{1}{Z}_{2}-\alpha \beta \zeta \sigma \phi {R}_{1}{Z}_{2}-\pi \beta \mu \nu {R}_{1}{\beta }_{1}-\pi \beta \mu \sigma {R}_{1}{\beta }_{1}+\pi \beta \zeta \mu \sigma {R}_{1}{\beta }_{1}+\pi \beta \mu \nu \sigma {R}_{1}{\beta }_{1}\right),$$

$${l}_{5}=-\pi \beta \nu \phi {R}_{1}{\beta }_{1}-\pi \beta \sigma \phi k\varepsilon {\delta }_{1}{R}_{1}{\beta }_{1}+\pi \beta \zeta \sigma \phi \left(1-k\right){\delta }_{1}{R}_{1}{\beta }_{1}+\pi \beta \nu \sigma \phi {R}_{1}{\beta }_{1}-\pi \alpha \beta \zeta \nu \sigma \phi (1-\varepsilon )k{\delta }_{1}{R}_{1}{\beta }_{1}+\beta \mu {Z}_{2}{\beta }_{1}+{\mu }^{2}{Z}_{2}{\beta }_{1}+\beta \phi \alpha {Z}_{2}{\beta }_{1}+\mu \varphi {Z}_{2}{\beta }_{1}+\pi \beta \mu \sigma \phi {\left(1-k\right){\delta }_{1}R}_{1}+\pi \beta \zeta \mu \sigma \phi {R}_{1}+\beta {\phi \mu }^{2}(1-\varepsilon )k{\delta }_{1}{Z}_{2}+{\mu }^{3}{\pi Z}_{2}+\beta \mu \alpha \phi {Z}_{2}+{\mu }^{2}\phi \alpha k\varepsilon {\delta }_{1}{Z}_{2}+\beta \mu \phi {R}_{1}{Z}_{2}+\beta \mu \sigma \phi {R}_{1}{Z}_{2}-\beta \phi \mu {R}_{1}{Z}_{2}+\beta \sigma \phi \alpha {k\varepsilon {\delta }_{1}R}_{1}{Z}_{2}+\alpha \beta \zeta \mu \sigma \phi {R}_{1}{Z}_{2}-\pi \beta \mu \nu \alpha \left(1-k\right){\delta }_{1}{R}_{1}{\beta }_{1}-\pi \beta \mu \phi \sigma {R}_{1}{\beta }_{1}+\pi \beta \zeta \alpha \mu \sigma {R}_{1}{\beta }_{1}+\pi \beta \mu \phi \nu \sigma {R}_{1}{\beta }_{1}+2\pi \beta \zeta \mu \sigma {R}_{1}-\beta \pi \alpha \phi {R}_{1}+\beta \pi \mu \sigma \phi k\varepsilon {\delta }_{1}{R}_{1}-\pi \beta \sigma \mu \varphi {R}_{1}+\pi \beta \zeta \mu \sigma \phi \left(1-k\right){\delta }_{1}{R}_{1}=0.$$

According to the Routh–Hurwitz criterion, for ${R}_{Eff}<1$, the Disease-free equilibrium ${D}^{0}$ is locally asymptotically stable if:

- $${l}_{0}>0,{l}_{1}>0,{l}_{2}>0,{l}_{3}>0,{l}_{4}>0,{l}_{5}>0.$$ Since
  $${l}_{0}=1>0$$
  $${l}_{1}=\left(\beta +3\mu +\phi +{Z}_{2}+{\beta }_{1}+k\varepsilon {\delta }_{1}\right)>0$$
  $${l}_{2}=\left(2\beta \mu +3{\mu }^{2}+\beta \phi +2\mu \phi -\beta {Z}_{1}+\beta \sigma {R}_{1}-\pi \beta \sigma {R}_{1}+\pi \beta \zeta \sigma {R}_{1}+3\mu {Z}_{2}+\varphi {Z}_{2}+\beta {\beta }_{1}+2\mu {\beta }_{1}+\varphi {\beta }_{1}+{Z}_{2}{\beta }_{1}\right)>0$$
  $${l}_{3}=\left(\beta {\mu }^{2}+{\mu }^{3}+\beta \mu \phi +{\mu }^{2}\phi -\beta \mu {R}_{1}+\beta \mu \sigma {R}_{1}-2\pi \beta \mu \sigma {R}_{1}+2\pi \beta \zeta \mu \sigma {R}_{1}-\beta \phi {R}_{1}+\beta \sigma \phi {R}_{1}-\pi \beta \sigma \phi {R}_{1}+\pi \beta \zeta \sigma \phi {R}_{1}-\left(1-\alpha \right)\beta \zeta \sigma \phi {R}_{1}+2\beta \mu {Z}_{2}+2\mu \phi {Z}_{2}+\left(1-k\right){\delta }_{1}+\beta \mu {\beta }_{1}+{\mu }^{2}{\beta }_{1}+\beta \phi {\beta }_{1}+\mu \phi {\beta }_{1}-\pi \beta \nu {R}_{1}{\beta }_{1}-\pi \beta \sigma {R}_{1}{\beta }_{1}+\pi \beta \zeta \sigma {R}_{1}{\beta }_{1}-\beta {R}_{1}{Z}_{2}\right)>0$$
  $${l}_{4}=\left(\beta \sigma {R}_{1}{Z}_{2}+\beta \phi {Z}_{2}+\pi \beta \nu \sigma {R}_{1}{\beta }_{1}+\beta {Z}_{2}{\beta }_{1}+2\mu {Z}_{2}{\beta }_{1}+\phi {Z}_{2}{\beta }_{1}+3{\mu }^{2}{Z}_{2}-\pi \beta {\mu }^{2}\sigma {R}_{1}+\pi \beta \zeta {\mu }^{2}\sigma {R}_{1}-\pi \beta \mu \sigma \phi {R}_{1}+\pi \beta \zeta \mu \sigma \phi {R}_{1}+\beta {\mu }^{2}{Z}_{2}+{\mu }^{3}{Z}_{2}+\beta \mu \phi {Z}_{2}+{\mu }^{2}\phi {Z}_{2}-\beta \mu {R}_{1}{Z}_{2}++\beta \mu \sigma {R}_{1}{Z}_{2}+\beta \phi {R}_{1}{Z}_{2}+\beta \sigma \phi {R}_{1}{Z}_{2}-\left(1-\alpha \right)\beta \zeta \sigma \phi {R}_{1}{Z}_{2}-\pi \beta \mu \nu {R}_{1}{\beta }_{1}-\pi \beta \mu \sigma {R}_{1}{\beta }_{1}+\pi \beta \zeta \mu \sigma {R}_{1}{\beta }_{1}+\pi \beta \mu \nu \sigma {R}_{1}{\beta }_{1}\right)>0$$
  $${l}_{5}=\frac{\left(\pi \beta \zeta \mu \sigma \phi \right)\left(\mu +\left(\left(1-k\right){\delta }_{1}\right)\left(1-\alpha \right)\beta \right)\left(\beta \phi +\pi \beta \mu \nu \left(1-\alpha \right)\right)}{\left(2\mu \phi {Z}_{2}+k\varepsilon {\delta }_{1}\beta \mu {\beta }_{1}\right)\left(\theta \beta +\phi \sigma \left(1-\alpha \right)\right)\left(\left((1-\varepsilon )k{\delta }_{1}\right)\left(1-\alpha \right)\pi \beta \zeta \mu \sigma +\beta \zeta \rho \phi \right)}\left(1-{R}_{Eff}\right).$$
  Here, $${l}_{5}>0$$ if $${R}_{Eff}<1.$$
  So, it is valid.
- ${l}_{1}{l}_{2}{l}_{3}>{{l}_{3}}^{2}+{{l}_{1}}^{2}{l}_{4}$ which is also valid.
- $\left({l}_{1}{l}_{4}-{l}_{5}\right)\left({l}_{1}{l}_{2}{l}_{3}-{{l}_{3}}^{2}-{{l}_{1}}^{2}{l}_{4}\right){>l}_{5}\left({l}_{1}{l}_{2}-{{l}_{3}}^{2}\right)+{l}_{1}{{l}_{5}}^{2}$ which is also true.

Since conditions (a), (b) and (c) are valid and

$${l}_{5}=\frac{\left(\pi \beta \zeta \mu \sigma \varphi \right)\left(\mu +\left(1-\alpha \right)\beta \right)\left(\beta \varphi +\pi \beta \mu \nu \left(1-\alpha \right)\right)}{\left(2\mu \varphi {Z}_{2}++\beta \mu {\beta }_{1}\right)\left(\theta \beta +\varphi \sigma \left(1-\alpha \right)\right)\left(\left(1-\alpha \right)\pi \beta \zeta \mu \sigma +\beta \zeta \rho \varphi \right)}\left(1-{R}_{Eff}\right)>0$$

for ${R}_{Eff}<1,$ then disease-free equilibrium point of our model is locally stable.

### Globally asymptotically stability of the DFEP (${D}^{0}$)

#### Theorem 4

*The disease-free equilibrium point of the system is globally asymptotically stable if* ${R}_{Eff}\le 1$ *and* $S={S}^{0}$ *and unstable for* ${R}_{Eff}>1.$

#### *Proof*

Let us define the Lyapunov function $L:{R}_{+}^{9}\to {R}_{+}$ s by:

$$L\left(S,V,E,Q,M,A,C,T,R\right)={d}_{1}\left(S-{S}^{0}\right)+{d}_{2}\left(V-{V}^{0}\right)+{d}_{3}E+{d}_{4}Q+{d}_{5}M+{d}_{6}A+{d}_{7}C+{d}_{8}T+{d}_{9}R$$

Differentiating the above equation gives us:

$${L}^{^{\prime}}={d}_{1}{S}^{^{\prime}}+{d}_{2}{V}^{^{\prime}}+{d}_{3}{E}^{^{\prime}}+{d}_{4}{Q}^{^{\prime}}+{d}_{5}{M}^{^{\prime}}+{d}_{6}{A}^{^{\prime}}+{d}_{7}{C}^{^{\prime}}+{d}_{8}{T}^{^{\prime}}+{d}_{9}{R}^{^{\prime}}$$

$$\begin{aligned} {L}^{^{\prime}} & ={d}_{1}\left(b\left(1-\omega \right)\left(1-\gamma C\right)+\tau V-\left(\lambda +\theta +\mu \right)S \right)+{d}_{2}\left(b\omega +\theta S-\left(\tau +\mu \right)V \right)+{d}_{3}\left(\lambda S+\left(1-k\right){\delta }_{1}M-\left(\beta +\mu \right)E \right) \\ & \quad +{d}_{4}\left(\zeta \sigma \beta E+k\varepsilon {\delta }_{1}M-\left(\phi +\mu \right)Q\right)+{d}_{5}\left(-\left({\delta }_{1}+\mu \right)M\right)+{d}_{6}\left(\left(1-\sigma \right)\beta E+\left(1-\varepsilon \right)k{\delta }_{1}M+\left(1-\alpha \right)\phi Q-\left({\beta }_{1}+\mu \right)A\right) \\ & \quad +{d}_{7}\left(b\left(1-\omega \right)\gamma C+\left(1-\zeta \right)\sigma \beta E+\nu {\beta }_{1}A-\left(\mu +d+\eta \right)C\right)+{d}_{8}\left(\eta C-\left(\delta +\mu \right)T\right)+{d}_{9}\left(\left(1-\nu \right){\beta }_{1}A+\delta T+\alpha \phi Q-\mu R\right) \end{aligned}$$

Choosing the constants ${d}_{1}={d}_{2}={d}_{3}=\zeta \sigma \beta ,{d}_{4}=\left(\beta +\mu \right),{d}_{5}=\frac{\left(1-k\right){\delta }_{1}\zeta \sigma \beta }{\left({\delta }_{1}+\mu \right)},{d}_{6}=\frac{\left(\phi +\mu \right)\left(\beta +\mu \right)}{\left(1-\alpha \right)\phi },$

$${d}_{7}=\zeta \left(\beta +\mu \right),{d}_{8}=\frac{\beta \left(1-\sigma \right)\left(\phi +\mu \right)\left(\beta +\mu \right)}{\left(\delta +\mu \right)},{d}_{9}=\frac{\mu \zeta \left(\beta +\mu \right)\beta \left(1-\sigma \right)\left(\phi +\mu \right)}{\left(1-\varepsilon \right)k{\delta }_{1}\left(\left(1-\nu \right){\beta }_{1}+\delta +\alpha \phi \right)}.$$

After arrangement and simplification,

$$\frac{dL}{dt}=\left(\frac{\beta \left(1-\sigma \right)\left(\phi +\mu \right)\left(\beta +\mu \right)}{\left(\delta +\mu \right)\left(\beta +\mu \right)}\right)\left(\frac{b\rho \beta \left(\tau -\mu \left(1-\omega \right)\right)\left[\left(\mu \left(1-\sigma \right)+\varphi \left(1-\sigma \left(1+\left(1-\alpha \right)\zeta \right)\right)\right)\left(\mu +d+\eta -b\gamma \left(1-\omega \right)+\theta \nu {\beta }_{1}\right)+\sigma (1-\zeta )(\mu +\phi )(\mu +{\beta }_{1})\right]}{\mu \left(\theta +\tau +\mu \right)(\beta +\mu )(\mu +\phi )(\mu +{\beta }_{1})\left(\mu +d+\eta -b\gamma \left(1-\omega \right)\right)}-1\right)$$

$$\frac{dL}{dt}=\left(\frac{\beta \left(1-\sigma \right)\left(\varphi +\mu \right)\left(\beta +\mu \right)}{\left(\delta +\mu \right)\left(\beta +\mu \right)}\right)\left({R}_{Eff}-1\right)$$

$\frac{dL}{dt}=0$ if $S={S}^{0}$, $V={V}^{0},E=Q=M=A=C=T=0$. On the other hand, $\frac{dL}{dt}\le 0$ for $S\le {S}^{0}$ and ${R}_{Eff}\le 1.$

Hence, disease free equilibrium point of our model is globally asymptotically stable.

### Endemic equilibrium point $\left({{\varvec{E}}}^{1}\right)$

We calculated endemic equilibrium point of our HBV model by equating the system of differential equation on (1) to zero.

Then, we get: ${E}^{1}=\left({S}^{*},{V}^{*},{E}^{*},{Q}^{*},{M}^{*},{A}^{*},{C}^{*},{T}^{*},{R}^{*}\right)$ where:

$${S}^{*} =\frac{{\left(b\left(1-\omega \right)\left(1-\gamma \right)\right)}^{2}\left(\left({\beta }_{1}+\mu \right)\left(\phi +\mu \right)\left(1-\zeta \right)\sigma \beta +\nu {\beta \beta }_{1}\left(\left(\phi +\mu \right)\left(1-\sigma \right)+\left(1-\alpha \right)\phi \zeta \sigma \right)\right)}{H}\left(1-\frac{1}{{R}_{Eff}}\right).$$

$${V}^{*}=\frac{b\omega H+\theta {\left(b\left(1-\omega \right)\left(1-\gamma \right)\right)}^{2}\left(\left({\beta }_{1}+\mu \right)\left(\varphi +\mu \right)\left(1-\zeta \right)\sigma \beta +\nu {\beta \beta }_{1}\left(\left(\varphi +\mu \right)\left(1-\sigma \right)+\left(1-\alpha \right)\varphi \zeta \sigma \right)\right)}{\left(\tau +\mu \right)H}\left(1-\frac{1}{{R}_{Eff}}\right)$$

$${E}^{*}=\frac{b\left(1-\omega \right)\left(1-\gamma \right)\left(\mu +d+\eta -b\gamma \left(1-\omega \right)\right)}{\left(\left(\phi +\mu \right)\left(1-\sigma \right)+\left(1-\alpha \right)\phi \zeta \sigma \right)}\left(1-\frac{1}{{R}_{Eff}}\right).$$

$${Q}^{*}=\frac{\zeta \sigma \beta \left(b\left(1-\omega \right)\left(1-\gamma \right)\left(\mu +d+\eta -b\gamma \left(1-\omega \right)\right)\right)}{\left(\phi +\mu \right)\left(\left(\phi +\mu \right)\left(1-\sigma \right)+\left(1-\alpha \right)\phi \zeta \sigma \right)}\left({R}_{Eff}-1\right).$$

$${A}^{*}=\frac{\left(1-\alpha \right)b\beta \left(\left(\phi +\mu \right)+\phi \sigma \beta \right)\left(1-\omega \right)\left(1-\gamma \right)\left(\mu +d+\eta -b\gamma \left(1-\omega \right)\right)}{\left(\phi +\mu \right)\left({\beta }_{1}+\mu \right)\left(\left(\phi +\mu \right)\left(1-\sigma \right)+\left(1-\alpha \right)\phi \zeta \sigma \right)}\left(1-\frac{1}{{R}_{Eff}}\right).$$

$${C}^{*}=\frac{\left(\left({\beta }_{1}+\mu \right)\left(1-\zeta \right)\sigma \beta +\nu {\beta \beta }_{1}\left(\left(\phi +\mu \right)\left(1-\sigma \right)+\left(1-\alpha \right)\phi \zeta \sigma \right)\right)\left(b\left(1-\omega \right)\left(1-\gamma \right)\right)}{\left({\beta }_{1}+\mu \right)\left(\left(\phi +\mu \right)\left(1-\sigma \right)+\left(1-\alpha \right)\phi \zeta \sigma \right)}\left(1-\frac{1}{{R}_{Eff}}\right).$$

$${T}^{*}=\frac{\eta \left(\left({\beta }_{1}+\mu \right)\left(1-\zeta \right)\sigma \beta +\nu {\beta \beta }_{1}\left(\left(\phi +\mu \right)\left(1-\sigma \right)+\left(1-\alpha \right)\phi \zeta \sigma \right)\right)\left(b\left(1-\omega \right)\left(1-\gamma \right)\right)}{\left({\beta }_{1}+\mu \right)\left(\left(\phi +\mu \right)\left(1-\sigma \right)+\left(1-\alpha \right)\phi \zeta \sigma \right)}\left(1-\frac{1}{{R}_{Eff}}\right).$$

where $$H=\left(\phi +\mu \right)\left({\beta }_{1}+\mu \right)\left(\mu +d+\eta -b\gamma \left(1-\omega \right)\right)\left(\rho \left(\left(\phi +\mu \right)\left(1-\sigma \right)\beta +\alpha \phi \zeta \sigma \beta \right)+\pi \left(\left({\beta }_{1}+\mu \right)\left(\phi +\mu \right)\left(1-\zeta \right)\sigma \beta +\nu {\beta \beta }_{1}\left(\left(\phi +\mu \right)\left(1-\sigma \right)+\left(1-\alpha \right)\phi \zeta \sigma \right)\right)+\left(\theta +\mu \right)\left(\phi +\mu \right)\left({\beta }_{1}+\mu \right)\right)$$.

2 $${R}^{*}=\left[\frac{\begin{array}{c}\left(1-\nu \right){\beta }_{1}\left(\left(1-\alpha \right)b\beta \left(\left(\phi +\mu \right)+\phi \sigma \beta \right)\left(1-\omega \right)\left(1-\gamma \right)\left(\mu +d+\eta -b\gamma \left(1-\omega \right)\right)\right)\\ +\delta \eta \left(\phi +\mu \right)\left(\left({\beta }_{1}+\mu \right)\left(1-\zeta \right)\sigma \beta +\nu {\beta \beta }_{1}\left(\left(\phi +\mu \right)\left(1-\sigma \right)+\left(1-\alpha \right)\phi \zeta \sigma \right)\right)\left(b\left(1-\omega \right)\left(1-\gamma \right)\right)\\ +\alpha \phi \zeta \sigma \beta \left({\beta }_{1}+\mu \right)b\left(1-\omega \right)\left(1-\gamma \right)\left(\mu +d+\eta -b\gamma \left(1-\omega \right)\right)\end{array}}{{\varvec{\mu}}\left(\left(\phi +\mu \right)\left({\beta }_{1}+\mu \right)\left(\left(\phi +\mu \right)\left(1-\sigma \right)+\left(1-\alpha \right)\phi \zeta \sigma \right)\right)}\right]\left(1-\frac{1}{{R}_{Eff}}\right)$$

### Uniqueness of endemic equilibrium point

#### **Lemma 1**

*A unique endemic equilibrium point* ${E}^{1}$ *exist for* ${R}_{Eff}>1,$ *and no endemic equilibrium otherwise.*

#### *Proof*

The system (1) has a unique endemic equilibrium point if ${R}_{Eff}>1.$

Endemic equilibrium of the system is given by:

$${S}^{*} =\frac{{\left(b\left(1-\omega \right)\left(1-\gamma \right)\right)}^{2}\left(\left({\beta }_{1}+\mu \right)\left(\phi +\mu \right)\left(1-\zeta \right)\sigma \beta +\nu {\beta \beta }_{1}\left(\left(\phi +\mu \right)\left(1-\sigma \right)+\left(1-\alpha \right)\phi \zeta \sigma \right)\right)}{H}\left(1-\frac{1}{{R}_{Eff}}\right).$$

$${V}^{*}=\frac{b\omega H+\theta {\left(b\left(1-\omega \right)\left(1-\gamma \right)\right)}^{2}\left(\left({\beta }_{1}+\mu \right)\left(\varphi +\mu \right)\left(1-\zeta \right)\sigma \beta +\nu {\beta \beta }_{1}\left(\left(\varphi +\mu \right)\left(1-\sigma \right)+\left(1-\alpha \right)\varphi \zeta \sigma \right)\right)}{\left(\tau +\mu \right)H}\left(1-\frac{1}{{R}_{Eff}}\right)$$

$${E}^{*}=\frac{b\left(1-\omega \right)\left(1-\gamma \right)\left(\mu +d+\eta -b\gamma \left(1-\omega \right)\right)}{\left(\left(\phi +\mu \right)\left(1-\sigma \right)+\left(1-\alpha \right)\phi \zeta \sigma \right)}\left(1-\frac{1}{{R}_{Eff}}\right).$$

$${Q}^{*}=\frac{\zeta \sigma \beta \left(b\left(1-\omega \right)\left(1-\gamma \right)\left(\mu +d+\eta -b\gamma \left(1-\omega \right)\right)\right)}{\left(\phi +\mu \right)\left(\left(\phi +\mu \right)\left(1-\sigma \right)+\left(1-\alpha \right)\phi \zeta \sigma \right)}\left({R}_{Eff}-1\right).$$

$${A}^{*}=\frac{\left(1-\alpha \right)b\beta \left(\left(\phi +\mu \right)+\phi \sigma \beta \right)\left(1-\omega \right)\left(1-\gamma \right)\left(\mu +d+\eta -b\gamma \left(1-\omega \right)\right)}{\left(\phi +\mu \right)\left({\beta }_{1}+\mu \right)\left(\left(\phi +\mu \right)\left(1-\sigma \right)+\left(1-\alpha \right)\phi \zeta \sigma \right)}\left(1-\frac{1}{{R}_{Eff}}\right).$$

$${C}^{*}=\frac{\left(\left({\beta }_{1}+\mu \right)\left(1-\zeta \right)\sigma \beta +\nu {\beta \beta }_{1}\left(\left(\phi +\mu \right)\left(1-\sigma \right)+\left(1-\alpha \right)\phi \zeta \sigma \right)\right)\left(b\left(1-\omega \right)\left(1-\gamma \right)\right)}{\left({\beta }_{1}+\mu \right)\left(\left(\phi +\mu \right)\left(1-\sigma \right)+\left(1-\alpha \right)\phi \zeta \sigma \right)}\left(1-\frac{1}{{R}_{Eff}}\right).$$

$${T}^{*}=\frac{\eta \left(\left({\beta }_{1}+\mu \right)\left(1-\zeta \right)\sigma \beta +\nu {\beta \beta }_{1}\left(\left(\phi +\mu \right)\left(1-\sigma \right)+\left(1-\alpha \right)\phi \zeta \sigma \right)\right)\left(b\left(1-\omega \right)\left(1-\gamma \right)\right)}{\left({\beta }_{1}+\mu \right)\left(\left(\phi +\mu \right)\left(1-\sigma \right)+\left(1-\alpha \right)\phi \zeta \sigma \right)}\left(1-\frac{1}{{R}_{Eff}}\right).$$

where $$H=\left(\phi +\mu \right)\left({\beta }_{1}+\mu \right)\left(\mu +d+\eta -b\gamma \left(1-\omega \right)\right)\left(\rho \left(\left(\phi +\mu \right)\left(1-\sigma \right)\beta +\alpha \phi \zeta \sigma \beta \right)+\pi \left(\left({\beta }_{1}+\mu \right)\left(\phi +\mu \right)\left(1-\zeta \right)\sigma \beta +\nu {\beta \beta }_{1}\left(\left(\phi +\mu \right)\left(1-\sigma \right)+\left(1-\alpha \right)\phi \zeta \sigma \right)\right)+\left(\theta +\mu \right)\left(\phi +\mu \right)\left({\beta }_{1}+\mu \right)\right)$$.

$${R}^{*}=\left[\frac{\begin{array}{c}\left(1-\nu \right){\beta }_{1}\left(\left(1-\alpha \right)b\beta \left(\left(\phi +\mu \right)+\phi \sigma \beta \right)\left(1-\omega \right)\left(1-\gamma \right)\left(\mu +d+\eta -b\gamma \left(1-\omega \right)\right)\right)\\ +\delta \eta \left(\phi +\mu \right)\left(\left({\beta }_{1}+\mu \right)\left(1-\zeta \right)\sigma \beta +\nu {\beta \beta }_{1}\left(\left(\phi +\mu \right)\left(1-\sigma \right)+\left(1-\alpha \right)\phi \zeta \sigma \right)\right)\left(b\left(1-\omega \right)\left(1-\gamma \right)\right)\\ +\alpha \phi \zeta \sigma \beta \left({\beta }_{1}+\mu \right)b\left(1-\omega \right)\left(1-\gamma \right)\left(\mu +d+\eta -b\gamma \left(1-\omega \right)\right)\end{array}}{{\varvec{\mu}}\left(\left(\phi +\mu \right)\left({\beta }_{1}+\mu \right)\left(\left(\phi +\mu \right)\left(1-\sigma \right)+\left(1-\alpha \right)\phi \zeta \sigma \right)\right)}\right]\left(1-\frac{1}{{R}_{Eff}}\right)$$

where force of infection of EEP is:

3 $$\begin{aligned} {\lambda }^{*} & =\rho \left({A}^{*}+\pi {C}^{*}\right) \\ &=\rho \left(\frac{\left(1-\alpha \right)b\beta \left(\left(\phi +\mu \right)+\phi \sigma \beta \right)\left(1-\omega \right)\left(1-\gamma \right)\left(\mu +d+\eta -b\gamma \left(1-\omega \right)\right)}{\left(\phi +\mu \right)\left({\beta }_{1}+\mu \right)\left(\left(\phi +\mu \right)\left(1-\sigma \right)+\left(1-\alpha \right)\phi \zeta \sigma \right)}\left(1-\frac{1}{{R}_{Eff}}\right) +{\varvec{\pi}}\frac{\left(\left({\beta }_{1}+\mu \right)\left(1-\zeta \right)\sigma \beta +\nu {\beta \beta }_{1}\left(\left(\phi +\mu \right)\left(1-\sigma \right)+\left(1-\alpha \right)\phi \zeta \sigma \right)\right)\left(b\left(1-\omega \right)\left(1-\gamma \right)\right)}{\left({\beta }_{1}+\mu \right)\left(\left(\phi +\mu \right)\left(1-\sigma \right)+\left(1-\alpha \right)\phi \zeta \sigma \right)}\left(1-\frac{1}{{R}_{Eff}}\right)\right) \\ & =\rho \left(\frac{\begin{array}{c}\left(1-\alpha \right)b\beta \left(\left(\phi +\mu \right)+\phi \sigma \beta \right)\left(1-\omega \right)\left(1-\gamma \right)\left(\mu +d+\eta -b\gamma \left(1-\omega \right)\right)\\ +\pi \left(\phi +\mu \right)\left(\left({\beta }_{1}+\mu \right)\left(1-\zeta \right)\sigma \beta +\nu {\beta \beta }_{1}\left(\left(\phi +\mu \right)\left(1-\sigma \right)+\left(1-\alpha \right)\phi \zeta \sigma \right)\right)\left(b\left(1-\omega \right)\left(1-\gamma \right)\right)\end{array}}{\left(\phi +\mu \right)\left({\beta }_{1}+\mu \right)\left(\left(\phi +\mu \right)\left(1-\sigma \right)+\left(1-\alpha \right)\phi \zeta \sigma \right)}\right)\left(1-\frac{1}{{R}_{Eff}}\right) \end{aligned}$$

After rearranging and simplifying (3), we get:

Since $$\mathrm{W}=\left(\frac{\begin{array}{c}\left(1-\mathrm{\alpha }\right)b\beta \left(\left(\upphi +\upmu \right)+\mathrm{\phi \sigma \beta }\right)\left(1-\upomega \right)\left(1-\upgamma \right)\left(\upmu +\mathrm{d}+\upeta -\mathrm{b\gamma }\left(1-\upomega \right)\right)\\ +\pi \left(\upphi +\upmu \right)\left(\left({\upbeta }_{1}+\upmu \right)\left(1-\upzeta \right)\mathrm{\sigma \beta }+\upnu {\mathrm{\beta \beta }}_{1}\left(\left(\upphi +\upmu \right)\left(1-\upsigma \right)+\left(1-\mathrm{\alpha }\right)\mathrm{\phi \zeta \sigma }\right)\right)\left(\mathrm{b}\left(1-\upomega \right)\left(1-\upgamma \right)\right)\end{array}}{\left(\upphi +\upmu \right)\left({\upbeta }_{1}+\upmu \right)\left(\left(\upphi +\upmu \right)\left(1-\upsigma \right)+\left(1-\mathrm{\alpha }\right)\mathrm{\phi \zeta \sigma }\right)}\right)>0,$$ then, ${\uplambda }^{*}>0$ if $\left(1-\frac{1}{{\mathrm{R}}_{\mathrm{Eff}}}\right)>0$ which implies ${\mathrm{R}}_{\mathrm{Eff}}>1.$

Thus, a unique endemic equilibrium exists when ${\mathrm{R}}_{\mathrm{Eff}}>1.$

### Local stability of endemic equilibrium point

#### Theorem 5

*The endemic equilibrium point* $\left({E}^{1}\right)$ *is locally asymptotically stable if* ${\mathrm{R}}_{\mathrm{Eff}}>1$.

#### *Proof*

The Jacobean matrix of the system (1) is given by:

$$J\left({D}^{*}\right)=\left[\begin{array}{ccccccccc}-f& \tau & 0& 0& 0& -{Z}_{1}& -{A}_{7}& 0& 0\\ \theta & -{A}_{1}& 0& 0& 0& 0& 0& 0& 0\\ e& 0& -{A}_{2}& 0& {A}_{10}& {Z}_{1}& \pi {Z}_{1}& 0& 0\\ 0& 0& \zeta \sigma \beta & -{A}_{3}& k\varepsilon {\delta }_{1}& 0& 0& 0& 0\\ 0& 0& 0& 0& -{A}_{4}& 0& 0& 0& 0\\ 0& 0& {A}_{8}& (1-\alpha )\phi & {A}_{11}& -{A}_{5}& 0& 0& 0\\ 0& 0& {A}_{9}& 0& 0& \nu {\beta }_{1}& -{Z}_{2}& 0& 0\\ 0& 0& 0& 0& 0& 0& \eta & -{A}_{6}& 0\\ 0& 0& 0& \mathrm{\alpha }\phi & 0& (1-\nu ){\beta }_{1}& 0& \delta & -\mu \end{array}\right]$$

where $$e=\rho \left({A}^{*}+\pi {C}^{*}\right),f=\lambda +\theta +\mu ,{Z}_{1}=\rho {S}^{0},{Z}_{2}=\left(\mu +d+\eta -b\gamma \left(1-\omega \right)\right),{A}_{1}=\left(\tau +\mu \right),{A}_{2}=\left(\beta +\mu \right),{A}_{3}=\left(\phi +\mu \right),{A}_{4}=\left({\delta }_{1}+\mu \right),{A}_{5}=\left({\beta }_{1}+\mu \right),{A}_{6}=\left(\delta +\mu \right),{A}_{7}=\pi {Z}_{1}+b\gamma \left(1-\omega \right)\gamma ,{A}_{8}=\left(1-\sigma \right)\beta ,{A}_{9}=\left(1-\zeta \right)\sigma \beta ,{A}_{10}=\left(1-k\right){\delta }_{1},{A}_{11}=(1-\varepsilon )k{\delta }_{1}$$.

Then, determinant of the matrix is given by:

$$\left|\begin{array}{ccccccccc}-f-\lambda & \tau & 0& 0& 0& -{Z}_{1}& -{A}_{7}& 0& 0\\ \theta & -{A}_{1}-\lambda & 0& 0& 0& 0& 0& 0& 0\\ e& 0& -{A}_{2}-\lambda & 0& {A}_{10}& {Z}_{1}& \pi {Z}_{1}& 0& 0\\ 0& 0& \zeta \sigma \beta & -{A}_{3}-\lambda & k\varepsilon {\delta }_{1}& 0& 0& 0& 0\\ 0& 0& 0& 0& -{A}_{4}-\lambda & 0& 0& 0& 0\\ 0& 0& {A}_{8}& (1-\alpha )\phi & {A}_{11}& -{A}_{5}-\lambda & 0& 0& 0\\ 0& 0& {A}_{9}& 0& 0& \nu {\beta }_{1}& -{Z}_{2}-\lambda & 0& 0\\ 0& 0& 0& 0& 0& 0& \eta & -{A}_{6}-\lambda & 0\\ 0& 0& 0& \mathrm{\alpha }\phi & 0& (1-\nu ){\beta }_{1}& 0& \delta & -\mu -\lambda \end{array}\right|=0$$

The characteristic equation of the matrix is:

This implies:

$$(-\lambda -\mu )\left(-\delta -\lambda -\mu \right)\left(-\lambda -\mu -{\delta }_{1}\right)\left({a}_{0}{\lambda }^{6}+{a}_{1}{\lambda }^{5}+{a}_{2}{\lambda }^{4}+{a}_{3}{\lambda }^{3}+{a}_{4}{\lambda }^{2}+{a}_{5}\lambda +{a}_{6}\right)=0.$$

where:

$${a}_{0}=1,{a}_{1}=\left(f+{A}_{1}+{A}_{2}+{A}_{3}\right)>0,{a}_{2}=\left(-\theta +f{A}_{1}+f{A}_{2}+{A}_{1}{A}_{2}+{fA}_{3}+{A}_{1}{A}_{3}+{A}_{2}{A}_{3}+{A}_{5}\right)>0,{a}_{3}=\left(-\theta \tau \left({A}_{2}+{A}_{3}\right)-\pi \nu {A}_{8}{Z}_{1}{\beta }_{1}+f{{A}_{1}A}_{2}+{A}_{1}{A}_{2}+f{A}_{1}{A}_{3}+f{A}_{2}{A}_{3}+{{A}_{1}A}_{2}{A}_{3}\left(f+1\right)\right)>0,{a}_{4}=\left(-\theta \tau {A}_{2}{A}_{3}+{{fA}_{1}A}_{2}{A}_{3}+e\nu {A}_{7}{A}_{8}-\pi \alpha \beta \zeta \nu \sigma {Z}_{1}{\beta }_{1}-f\pi \nu {A}_{8}{Z}_{1}{\beta }_{1}-\pi \nu {A}_{3}{A}_{8}{Z}_{1}{\beta }_{1}-\pi \nu {A}_{1}{A}_{8}{Z}_{1}{\beta }_{1}\right)>0,{a}_{5}=\left(\left(1-\alpha \right)e\beta \phi \sigma \zeta \nu {A}_{7}+e\nu {A}_{1}{A}_{7}{A}_{8}{\beta }_{1}-\left(1-\alpha \right)f\pi \sigma \beta \phi \zeta \nu {Z}_{1}{\beta }_{1}-\left(1-\alpha \right)\pi \beta \sigma \phi \zeta \nu {{A}_{1}Z}_{1}{\beta }_{1}+\pi \theta \tau \nu {A}_{8}{Z}_{1}{\beta }_{1}-f\pi \nu {{A}_{1}{A}_{8}Z}_{1}{\beta }_{1}-f\pi \nu {{A}_{3}{A}_{8}Z}_{1}{\beta }_{1}-\pi \nu {{A}_{3}{A}_{8}Z}_{1}{\beta }_{1}\right)>0,{a}_{6}=\left(1-\alpha \right)\tau e\beta \zeta \nu \phi {Z}_{2}{A}_{2}{A}_{3}{A}_{5}-e\nu {A}_{2}{A}_{3}{A}_{5}{Z}_{2}+\left(1-\alpha \right){\tau e\beta Z}_{1}\left({A}_{8}+{A}_{9}{A}_{2}+{A}_{3}{A}_{5}{A}_{9}+f\pi \nu {A}_{1}{{A}_{3}{A}_{8}Z}_{1}{\beta }_{1}\right)>0.$$

Here, since $f\pi \nu {A}_{1}{{A}_{3}{A}_{8}Z}_{1}{\beta }_{1}$ is positive, we can simplify:

$${a}_{6}=\left(1-\alpha \right)\tau e\beta \zeta \nu \phi {Z}_{2}{A}_{2}{A}_{3}{A}_{5}+\left(1-\alpha \right)\tau e\beta \zeta \nu \phi {A}_{2}{A}_{3}{A}_{5}{Z}_{2}+\left(1-\alpha \right)\tau e\beta \zeta \nu \phi \left({A}_{8}+{A}_{9}{A}_{2}+{A}_{3}{A}_{5}{A}_{9}\right)>0.$$

$${a}_{6}=\tau e\alpha \beta \zeta \nu \phi \left(\rho \left(\begin{array}{c}\frac{\boldsymbol{\aleph }}{\mathcal{J}}\left(1-\frac{1}{{R}_{Eff}}\right)\\ +\pi \left(\frac{\mathcal{L}}{\mathcalligra{l}}\left(1-\frac{1}{{R}_{Eff}}\right). \right)\end{array}\right)\frac{\left({A}_{2}{A}_{3}{A}_{5}{Z}_{2}-{Z}_{1}\left({A}_{8}+{A}_{9}{A}_{2}+{A}_{3}{A}_{5}{A}_{9}\right)\right)}{\tau e\alpha \beta \zeta \nu \phi \left({A}_{2}{A}_{3}{A}_{5}{Z}_{2}\right)}-1\right)>0$$

where $$\frac{\boldsymbol{\aleph }}{\mathcal{J}}=\frac{\beta \left(b\omega -b\omega \gamma \right)\left(\mu +d+\eta -b\omega \gamma \right)}{\left(\phi +\mu \right)\left({\beta }_{1}+\mu \right)}$$

$$\frac{\mathcal{L}}{\mathcalligra{l}}=\frac{\left(b\omega -b\omega \gamma \right)\left(\left({\beta }_{1}+\mu \right)\left(\phi +\mu \right)\left(1-\zeta \right)\sigma \beta +\nu {\beta \beta }_{1}\left(\left(\phi +\mu \right)\left(1-\sigma \right)+\alpha \phi \zeta \sigma \right)\right)}{\left(\phi +\mu \right)\left({\beta }_{1}+\mu \right)\left(\left(\phi +\mu \right)\left(1-\sigma \right)+\alpha \phi \zeta \sigma \right)}$$

$${R}_{Eff}=\frac{\left({A}_{2}{A}_{3}{A}_{5}{Z}_{2}-{Z}_{1}\left({A}_{8}+{A}_{9}{A}_{2}+{A}_{3}{A}_{5}{A}_{9}\right)\right)}{\left({A}_{2}{A}_{3}{A}_{5}{Z}_{2}\right)}$$

In order for ${a}_{6}>0,$ ${R}_{Eff}>1.$

Here, from Routh-Hurwitz criteria ${b}_{1}=\frac{{a}_{4}{a}_{5}-{a}_{1}}{{a}_{5}}>0,{b}_{2}=\frac{{a}_{2}{a}_{5}-{a}_{1}}{{a}_{5}}>0,{b}_{3}=\frac{{a}_{4}{a}_{5}-{a}_{1}}{{a}_{5}}>0,{c}_{1}=\frac{{a}_{3}{b}_{1}-{a}_{5}{b}_{2}}{{b}_{1}}>0,{c}_{2}=\frac{{a}_{5}{b}_{3}-{a}_{1}{b}_{1}}{{b}_{1}}>0,{d}_{1}=\frac{{a}_{5}{b}_{3}-{a}_{1}{b}_{1}}{{b}_{1}}>0,$ and ${a}_{6}>0$ for ${R}_{Eff}>1$.

Hence, the first column of the Routh Hurwitz array has no sign change, and then the root of the characteristic equation of the dynamical system is negative. Therefore, we can conclude that Endemic Equilibrium point of our model is locally asymptotically stable.

### Global stability analysis of endemic equilibrium point

#### Theorem 6

*The equations of the model have a positive distinctive endemic equilibrium whenever* ${R }_{Eff}> 1$, *which is said to be globally asymptotically stable*.

#### *Proof*

To prove the global asymptotic stability of the endemic equilibrium, we use the method of Lyapunov functions. Considering the Lyapunov function defined as:

$$L\left({S}^{*},{V}^{*},{E}^{*},{Q}^{*},{A}^{*},{C}^{*},{T}^{*},{R}^{*}\right)=\left(S-{S}^{*}\mathrm{ln}\left(\frac{S}{{S}^{*}}\right)\right)+\left(V-{V}^{*}\mathrm{ln}\left(\frac{V}{{V}^{*}}\right)\right)+\left(E-{E}^{*}\mathrm{ln}\left(\frac{E}{{E}^{*}}\right)\right)+\left(Q-{Q}^{*}\mathrm{ln}\left(\frac{Q}{{Q}^{*}}\right)\right)+\left(A-{A}^{*}\mathrm{ln}\left(\frac{A}{{A}^{*}}\right)\right)+\left(C-{C}^{*}\mathrm{ln}\left(\frac{C}{{C}^{*}}\right)\right)+\left(T-{T}^{*}\mathrm{ln}\left(\frac{T}{{T}^{*}}\right)\right)+\left(R-{R}^{*}\mathrm{ln}\left(\frac{R}{{R}^{*}}\right)\right).$$

where *L* takes it derivative along the system directly as:

$$\frac{dL}{dt}=\left(1-\frac{{S}^{*}}{S}\right)\frac{dS}{dt}+\left(1-\frac{{V}^{*}}{V}\right)\frac{dV}{dt}+\left(1-\frac{{E}^{*}}{E}\right)\frac{dE}{dt}+\left(1-\frac{{Q}^{*}}{Q}\right)\frac{dQ}{dt}+\left(1-\frac{{A}^{*}}{A}\right)\frac{dA}{dt}+\left(1-\frac{{C}^{*}}{C}\right)\frac{dC}{dt}$$

$$\frac{dL}{dt}=\left(1-\frac{{S}^{*}}{S}\right)\left[b\left(1-\omega \right)\left(1-\gamma C\right)+\tau V-\left(\lambda +\theta +\mu \right)S \right]+\left(1-\frac{{V}^{*}}{V}\right)\left[b\omega +\theta S-\left(\tau +\mu \right)V\right]+\left(1-\frac{{E}^{*}}{E}\right)\left[\lambda S+\left(1-k\right){\delta }_{1}M-\left(\beta +\mu \right)E\right]+\left(1-\frac{{Q}^{*}}{Q}\right)\left[\zeta \sigma \beta E+k\varepsilon {\delta }_{1}M-\left(\phi +\mu \right)Q\right]+\left(1-\frac{{A}^{*}}{A}\right)\left[\left(1-\sigma \right)\beta E+\left(1-\varepsilon \right)k{\delta }_{1}M+\left(1-\alpha \right)\phi Q-\left({\beta }_{1}+\mu \right)A\right]+\left(1-\frac{{C}^{*}}{C}\right)\left[b\left(1-\omega \right)\gamma C+\left(1-\zeta \right)\sigma \beta E+\nu {\beta }_{1}A-\left(\mu +d+\eta \right)C\right]+\left(1-\frac{{T}^{*}}{T}\right)\left[\eta C-\left(\delta +\mu \right)T \right]+\left(1-\frac{{R}^{*}}{R}\right)\left[\left(1-\nu \right){\beta }_{1}A+\delta T+\alpha \phi Q-\mu R\right]$$.

At equilibrium point, ${\lambda }^{*}=\rho \left({A}^{*}+\pi {C}^{*}\right)$ and using (1), we get:

$$b\left(1-\omega \right)=b\gamma \left(1-\omega \right){C}^{*}+\tau {V}^{*}-\left(\rho {A}^{*}+\rho \pi {C}^{*}+\theta +\mu \right){S}^{*},$$

$$b\omega =\left(\tau +\mu \right){V}^{*}-\theta {S}^{*},$$

$$\left(\beta +\mu \right)=\frac{\lambda {S}^{*}}{{E}^{*}}+\frac{\left(1-k\right){\delta }_{1}{M}^{*}}{{E}^{*}},$$

$$\left(\phi +\mu \right)=\frac{\zeta \sigma \beta {E}^{*}}{{Q}^{*}}+\frac{k\varepsilon {\delta }_{1}{M}^{*}}{{Q}^{*}},$$

$$\left({\beta }_{1}+\mu \right)=\frac{\left(1-\sigma \right)\beta {E}^{*}+\left(1-\varepsilon \right)k{\delta }_{1}{M}^{*}+\left(1-\alpha \right)\phi {Q}^{*}}{{A}^{*}},$$

$$\left(\mu +d+\eta \right)=\frac{b\gamma \left(1-\omega \right){C}^{*}+\left(1-\zeta \right)\sigma \beta {E}^{*}+\nu {\beta }_{1}{A}^{*}}{{C}^{*}},$$

$$\left(\delta +\mu \right)=\frac{\eta {C}^{*}}{{T}^{*}},$$

$$\mu =\frac{\left(1-\nu \right){\beta }_{1}{A}^{*}+\delta {T}^{*}+\alpha \phi {Q}^{*}}{{R}^{*}}.$$

$$\dot{L}=\left(1-\frac{{S}^{*}}{S}\right)\left[b\left(1-\omega \right)\left(1-\gamma {C}^{*}\right)+\tau {V}^{*}-\left(\rho {A}^{*}+\rho \pi {C}^{*}+\theta +\mu \right){S}^{*}-\left(b\left(1-\omega \right)\left(1-\gamma C\right)+\tau V-\left(\rho A+\rho \pi C+\theta +\mu \right)\right)S\right]+\left(1-\frac{{V}^{*}}{V}\right)\left[b\omega +\theta {S}^{*}-\left(\tau +\mu \right){V}^{*}-\left(b\omega +\theta S-\left(\tau +\mu \right)V\right)\right]+\left(1-\frac{{E}^{*}}{E}\right)\left[\lambda S+\left(1-k\right){\delta }_{1}M-\left(\beta +\mu \right)E\right]+\left(1-\frac{{Q}^{*}}{Q}\right)\left[\zeta \sigma \beta {E}^{*}+\left(1-\varepsilon \right){\delta }_{1}{M}^{*}-\left(\varphi +\mu \right){Q}^{*}-\left(\zeta \sigma \beta E+\left(1-k\right){\delta }_{1}M-\left(\phi +\mu \right)Q\right)\right]+\left(1-\frac{{A}^{*}}{A}\right)\left[\left(1-\sigma \right)\beta {E}^{*}+\left(1-\varepsilon \right)k{\delta }_{1}{M}^{*}+\left(1-\alpha \right)\varphi {Q}^{*}-\left({\beta }_{1}+\mu \right)A-\left(\left(1-\sigma \right)\beta E+\left(1-k\right)\varepsilon {\delta }_{1}M+\left(1-\alpha \right)\phi Q-\left({\beta }_{1}+\mu \right)A\right)\right]+\left(1-\frac{{C}^{*}}{C}\right)\left[b\gamma \left(1-\omega \right){C}^{*}+\left(1-\zeta \right)\sigma \beta {E}^{*}+\nu {\beta }_{1}{A}^{*}-\left(\mu +d+\eta \right){C}^{*}-\left(b\gamma \left(1-\omega \right)C+\left(1-\zeta \right)\sigma \beta E+\nu {\beta }_{1}A-\left(\mu +d+\eta \right)C\right)\right]+\left(1-\frac{{T}^{*}}{T}\right)\eta {C}^{*}-\left(\delta +\mu \right){T}^{*}-\left(\eta C-\left(\delta +\mu \right)T\right)+\left(1-\frac{{R}^{*}}{R}\right)\left[\left(1-\nu \right){\beta }_{1}{A}^{*}+\delta {T}^{*}+\alpha \phi {Q}^{*}-\mu {R}^{*}-\left(\left(1-\nu \right){\beta }_{1}A+\delta T+\alpha \phi Q-\mu R\right)\right]$$.

After rearranging and simplification we get;

4 $$\dot{L}={P}_{2}-{P}_{1}$$

where:$${P}_{1}=b\gamma \left(1-\omega \right)C\left(1-\frac{{C}^{*}}{C}\right)\left(1-\frac{{S}^{*}}{S}\right)+\tau V\left(1-\frac{{V}^{*}}{V}\right)\left(1-\frac{{S}^{*}}{S}\right)+\rho AS\left(1-\frac{{S}^{*}{A}^{*}}{SA}\right)\left(1-\frac{{S}^{*}}{S}\right)+\rho \pi CS\left(1-\frac{{S}^{*}{C}^{*}}{SC}\right)\left(1-\frac{{S}^{*}}{S}\right),$$ $${P}_{2}=\theta S\left(1-\frac{{S}^{*}}{S}\right)\left(1-\frac{{V}^{*}}{V}\right)+\left(1-\frac{{E}^{*}}{E}\right)\left[\rho AS\left(1-\frac{{S}^{*}E}{S{E}^{*}}\right)+\rho \pi CS\left(1-\frac{{S}^{*}{E}^{*}}{SE}\right)+\left(1-k\right){\delta }_{1}M\left(1-\frac{{M}^{*}E}{M{E}^{*}}\right)\right]+\left(1-\frac{{Q}^{*}}{Q}\right)\left[\zeta \sigma \beta \left(1-\frac{Q{E}^{*}}{{EQ}^{*}}\right)+\left(1-k\right){\delta }_{1}M\left(1-\frac{{M}^{*}Q}{{MQ}^{*}}\right)\right]+\left(1-\frac{{A}^{*}}{A}\right)\left[\left(1-\sigma \right)\beta E\left(1-\frac{A{E}^{*}}{{EA}^{*}}\right)+\left(1-\varepsilon \right)k{\delta }_{1}M\left(1-\frac{A{M}^{*}}{{MA}^{*}}\right)+\left(1-\alpha \right)\phi Q\left(1-\frac{A{Q}^{*}}{{QA}^{*}}\right)\right]+\left(1-\frac{{C}^{*}}{C}\right)\left[b\gamma \left(1-\omega \right)\left(1-\frac{{C}^{*}}{C}\right)+\left(1-\zeta \right)\sigma \beta E\left(1-\frac{C{E}^{*}}{E{C}^{*}}\right)+\nu {\beta }_{1}A\left(1-\frac{{CA}^{*}}{A{C}^{*}}\right)\right]+\left(1-\frac{{T}^{*}}{T}\right)\eta C\left[\left(1-\frac{{C}^{*}T}{C{T}^{*}}\right) \right]+\left(1-\frac{{R}^{*}}{R}\right)\left[\left(1-\nu \right){\beta }_{1}A\left(1-\frac{{AR}^{*}}{{A}^{*}R}\right)+\delta T\left(1-\frac{{R}^{*}T}{R{T}^{*}}\right)+\alpha \phi Q\left(1-\frac{{R}^{*}Q}{R{Q}^{*}}\right)\right]$$

5 $$\dot{L}\le 0\mathrm{ if }{P}_{1}\ge {P}_{2}$$

$${P}_{1}\le 0$$ whenever $$SA\le {S}^{*}{A}^{*},SC\le {S}^{*}{C}^{*},{P}_{2}\le 0$$ whenever $${S}^{*}E\le S{E}^{*},{S}^{*}{E}^{*}\le SE,{M}^{*}E\le M{E}^{*},Q{E}^{*}\le {EQ}^{*},{M}^{*}Q\le {MQ}^{*},A{E}^{*}\le {EA}^{*},A{M}^{*}\le {MA}^{*},A{Q}^{*}\le {QA}^{*},C{E}^{*}\le E{C}^{*},{CA}^{*}\le A{C}^{*},{C}^{*}T\le C{T}^{*},{AR}^{*}\le {A}^{*}R,{R}^{*}T\le R{T}^{*},{R}^{*}Q\le R{Q}^{*}.$$

Thus, $\dot{L}\le 0$ if the conditions (4) and (5) holds.

Therefore, by LaSalle asymptotic stability theorem^36^, the positive equilibrium state $\frac{dL}{dt}\le 0$ is globally asymptotically stable in the positive region ${R}_{+}^{9}$ and if $\frac{dL}{dt}<0$ then, endemic equilibrium point is globally stable.

## Sensitivity analysis

Sensitivity analysis is defined as the study of how uncertainty in the output of a model can be attributed to different sources of uncertainty in the model input. Sensitivity analysis (SA) refers to a broad set of mathematical approaches designed to quantify how variation in model outputs may be attributed to model inputs. These approaches allow researchers to assess how much trust to put in results obtained from a particular mathematical model. The normalized forward sensitivity index of a variable ${R}_{Eff}$ that depends differentiable on a parameter $P$ is defined as: $SI\left(P\right)=\frac{\partial {R}_{Eff}}{\partial P}\times \frac{P}{{R}_{Eff}}$^34^. The parameter with higher sensitivity index magnitude is/are more influential than that with smaller magnitude of sensitive index. The sign of the sensitivity indices of ${\mathrm{R}}_{\mathrm{Eff}}$ with respect to the parameters show the positive or negative impact of the parameters. Here, we calculated sensitivity index for each parameter included in effective reproduction number using the parameters given on Table 1 above as follows.

**Figure 2:**
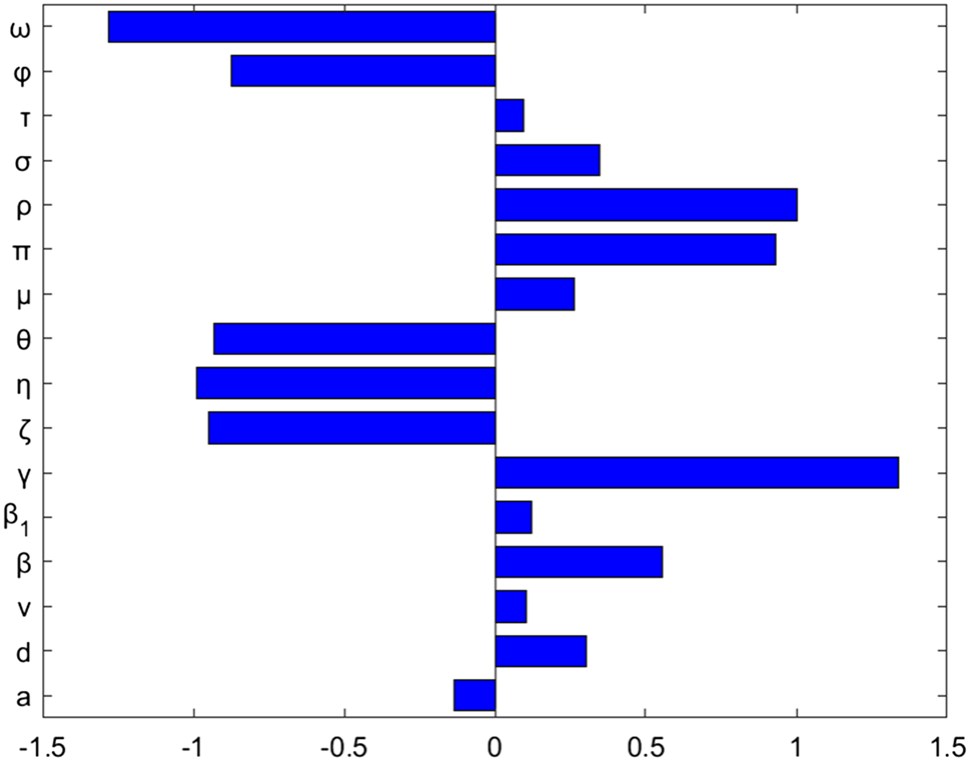
Sensitivity indices of the model parameters.

As it is shown in Fig. 2 above, perinatal infection rate, vaccination rate, contact rate, treatment rate, and screening rate of the exposed population are the first five most influential parameters in changing the values of effective reproduction number, and vaccination efficacy rate is the least influential parameter. Both perinatal infection rate and contact rate have a positive effect on effective reproduction numbers, whereas vaccination rate, treatment rate, and screening rate have a negative effect. Therefore, it is recommendable to boost those parameters having a negative effect and lessen those having a positive effect on the increment of the effective reproduction number.

**Figure 3:**
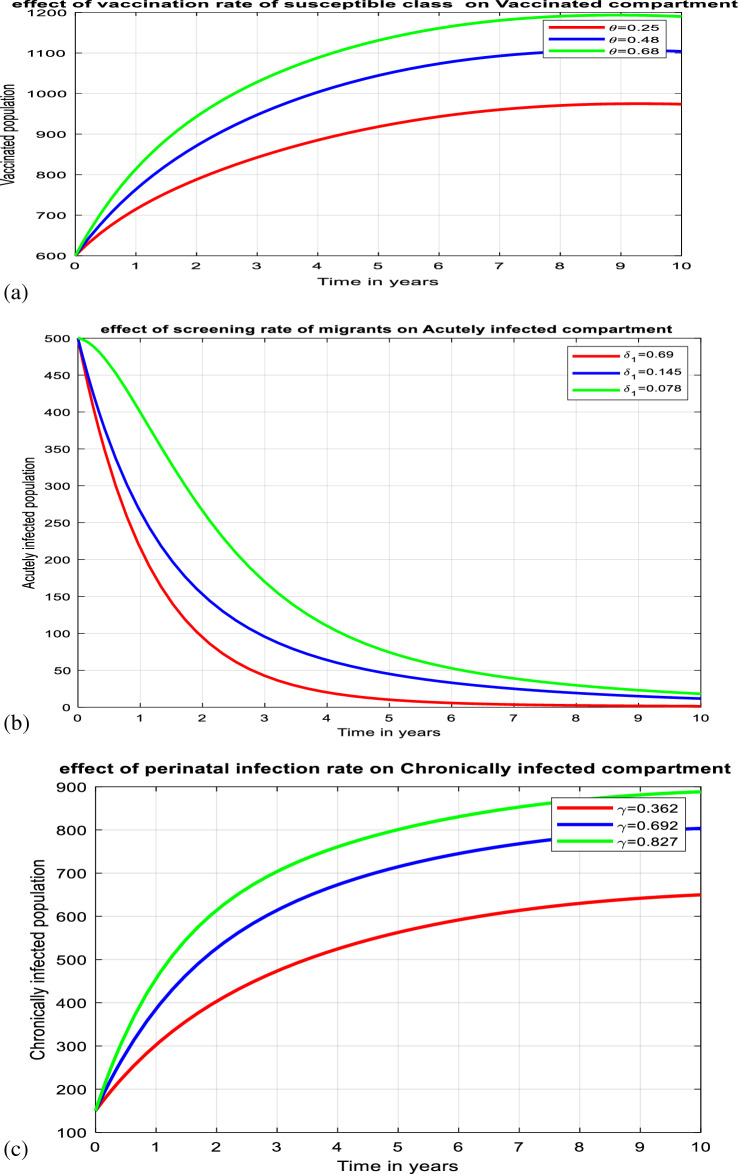

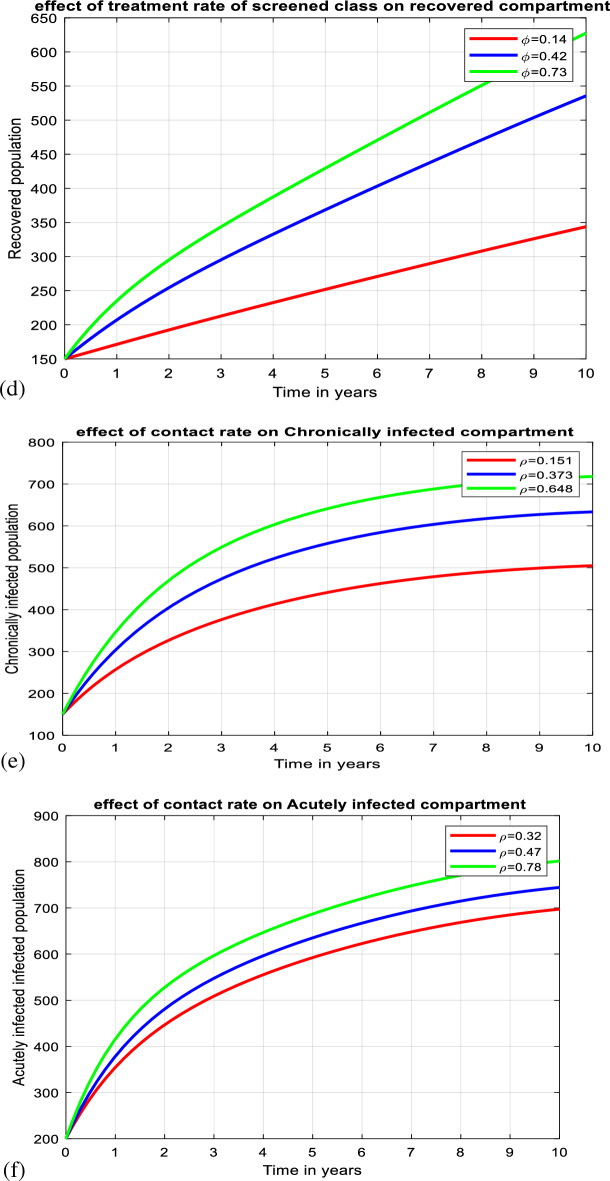

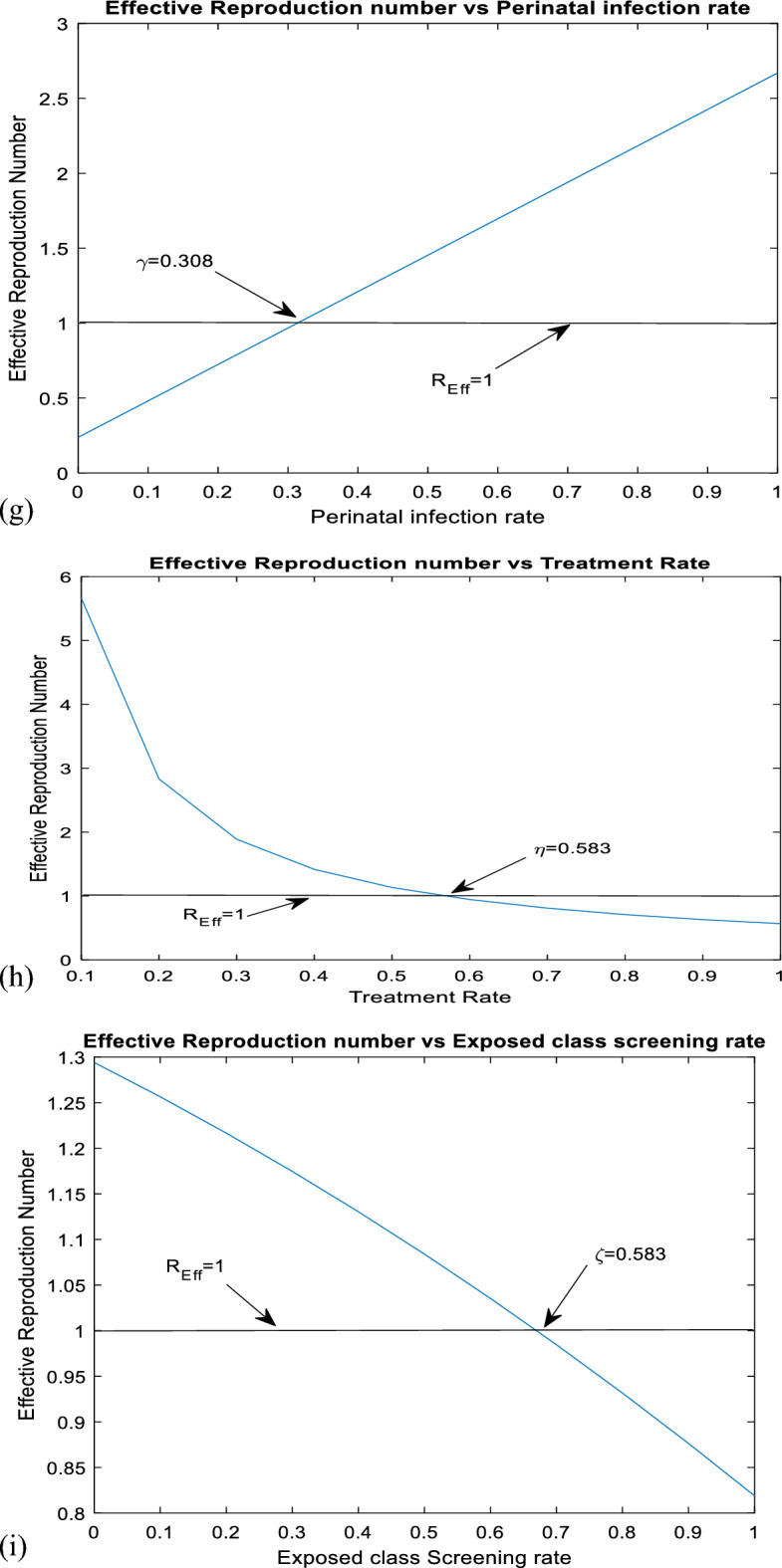

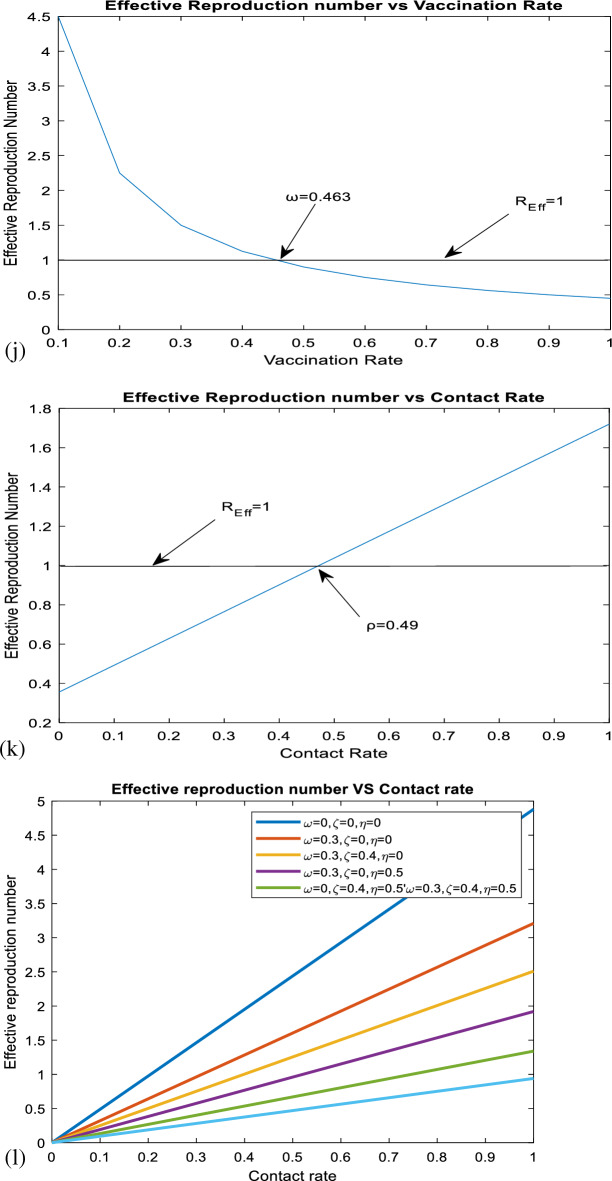
(**a**) Graph showing effect of vaccination of susceptible class on the vaccinated population. (**b**) Behavior of graph of Acutely infected class vs screening rate of migrants. (**c**) Behavior of perinatal infection rate versus chronically infective class. (**d**) Graph of treatment rate of screened class versus recovered class. (**e**) Effect of contact rate on acutely infected class. (**f**) Effect of contact rate on chronically infected class. (**g**) Graph of effective reproduction number vs perinatal infection rate. (**h**) Graph of Effective reproduction number in relation to treatment rate. (**i**) Graph of Effective reproduction number vs screening rate of HBV. (**j**) Behavior of Effective reproduction number vs vaccination rate (**k**) Behavior of Effective reproduction number in relation to contact rate of HBV. (**l**) Change in ${\mathrm{R}}_{\mathrm{Eff}}$ with change in intervention parameters versus contact rate.

According to numerous studies, the percentage of those who tested positive for anti-HBs antibodies 7–10 days, 1, 6, and 7 months after vaccination was 20.5%, 75.6%, 94.5%, and 99.2%, respectively. This finding explains why the early booster response predicts higher levels of protection at 1 and 6 months after vaccination. Figure 3a shows that subsequent vaccination of susceptible groups has a significant impact on increasing the number of immunized populations. As a result, vaccination has been identified as one of the most effective ways to prevent HBV transmission in the community.

Early detection and treatment rely heavily on screening. It can help prevent major illnesses, including cirrhosis and liver cancer, as well as slow the spread of infection. The advantage of screening migrants is that it detects people with asymptomatic chronic HBV infection acquired in their countries of origin who, if undiagnosed, are at a greater risk of developing HBV-related sequelae such as liver cirrhosis and hepatocellular carcinoma. Figure 3b shows that when the number of exposed classes decreases, the number of screening migrants increases. Then, screening is one of the measures that should be employed to control the spread of HBV in society.

Perinatal transmission is one way for HBV to be passed from mother to newborn during delivery. Perinatal HBV infection is strongly connected with HBeAg positivity in reproductive women and is associated with the greatest risk of developing chronic HBV infection, with 85% to 90% of babies born to HBeAg-positive mothers becoming chronic HBV carriers. Immunoprophylaxis for infants born to infected mothers includes the administration of hepatitis B vaccine and immune globulin within 12 h of birth. Figure 3c shows that when the rate of prenatal infection grows, so does the number of people who are chronically infected. This demonstrates that vertical transmission from a carrier mother to her infants increases the number of highly afflicted groups.

As a result, the best action is needed to cut off this transmission thread, which includes vaccinating newborns and providing early treatment to individuals who have developed viruses in their bodies. Since 2009, WHO has recommended that all infants receive 3–4 doses of HBV vaccine to prevent mother-to-child transmission, with the first dose provided as soon as possible after birth, preferably within 24 h.

Blood tests can detect symptoms of the hepatitis B virus in people's bodies and determine whether the infection is acute or chronic. The primary reason for screening for HBV is to reduce morbidity and mortality associated with liver disease and to prevent transmission. PEP is a treatment that can be utilized after suspected HBV exposure through intercourse, drug injecting equipment, or damage such as a needle stick injury. PEP is administered to reduce the chance of HBV infection. As shown in Fig. 3d, as the treatment rate of the screened class increases, so does the number of retrieved classes. This implies that successful treatment following screening contributes significantly to the increase in recovered patients.

Figure 3e demonstrates that when the contact rate between infected and uninfected people grows, so does the number of people who become infected. Hepatitis B is well recognized as being transferred when blood, sperm, or another body fluid from an infected individual enters the body of someone who is not infected with HBV. This can occur through sexual contact, the sharing of needles, syringes, or other drug-injection equipment, or transmission from mother to child. Then, Hepatitis B carriers should practice proper hygiene to avoid immediately exposing close contacts to their blood or other bodily fluids. Carriers should not share razors, toothbrushes, or any other item that could be contaminated with blood.

It is well recognized that anyone who lives with or has contact with someone who has chronic Hepatitis B should be checked. Hepatitis B is a dangerous condition that can be passed on from an infected person to other family and household members, caregivers, and sexual partners. Infants, in particular, are at high risk of becoming chronically infected with HBV if they come into contact with infected people. As a result, it is critical that all babies and unvaccinated children under the age of 19 avoid contact with HBV-infected adults. It is also recommended that, because sex is a typical route for HBV to spread from one person to another, partners refrain from having unprotected intercourse and instead use a condom or other latex barrier protection. As demonstrated in Fig. 3f, as the contact rate between HBV-infected persons and susceptible individuals increases, so does the number of chronically infected individuals.

Figure 3g illustrates that when the rate of perinatal infection rises, so does the number of effective reproductions. This demonstrates that perinatal infection significantly contributes to the spread of HBV illness throughout society. To reduce perinatal infection, expectant mothers should be vaccinated and treated with prophylaxis after delivery.

As shown in Fig. 3h, the effective reproduction number reduces as the chronically infected population's treatment rate rises. This implies that successful treatment reduces the spread of HBV infection in society. A group of prominent virologists, immunologists, and physicians specializing in viral hepatitis will use TherVacB, a newly created therapeutic vaccine, as an immunotherapy to cure HBV. TherVacB will be tested in a three-year clinical trial beginning in 2022 across Europe and Africa.

As shown in Fig. 3i above, screening of HBV-exposed individuals plays a great role in decreasing transmission of HBV disease after exposure by leading to early treatment, especially post-exposure prophylaxis. From this, we can understand that early screening of the exposed population and early treatment help suppress the progression of the exposed class to acute and chronic infective classes.

Figure 3j above shows that an increment in the vaccination rate results in a decrease in the transmission of HBV disease in society. Therefore, subsequent vaccination of the whole population is one of the most effective strategies for controlling the transmission of the virus. Keeping the vaccination rate above 0.463 enables us to suppress the transmission of the virus. Hepatitis B vaccination is highly effective in preventing healthcare-associated HBV infection and chronic infection among health care workers. Using mathematical models, it was estimated that HBV vaccination programs have prevented 210 million new HBV infections worldwide since their implementation.

In Fig. 3k above, contact rate contributes to increasing the effective reproduction number in society. Then, by decreasing the contact rate, it is possible to control the transmission of the virus in the community. By keeping the contact rate less than 0.49, we can make effective reproduction less than a unity.

HBV infection can be chronic, putting people at risk of death from cirrhosis and liver cancer. Vaccination is one of the most effective ways to prevent HBV transmission. It protects against Hepatitis B in 98% to 100% of cases. Several medications, including entecavir, tenofovir, lamivudine, adefovir, and telbivudine, can aid in the fight against the virus and slow its ability to harm the liver. Blood tests that measure HBV antigens and antibodies are used to screen for hepatitis B. The hepatitis surface antigen test detects the presence of HBV. It can also detect current infections, previous infections, and immunizations. In Fig. 3l above, we have shown the change in effective reproduction number in relation to the change in intervention parameters. It is shown that the effective reproduction number without intervention is equal to *4.88,* whereas the effective reproduction number with vaccination, treatment, and screening is equal to *0.94.* From this, we understand the effectiveness of the three intervention strategies in suppressing transmission of HBV by reducing the value of effective reproduction number. From this, one can understand that by applying screening to exposed and migrated classes, treating screened and chronically infected classes, vaccinating the susceptible classes subsequently, and maximizing these interventions rates, it is possible to change the endemic HBV into a non-endemic HBV by making Therefore, the combined effect of vaccination, treatment, and screening is visible in decreasing the transmission of HBV in society.

## Numerical simulation

The following is a discussion of the stability of both disease-free and endemic equilibrium points, the impacts of changing the most important parameters in each compartment of our model, and the relationship between the effective reproduction number and parameters used in the HBV model using MATLAB ode45.

**Figure 4:**
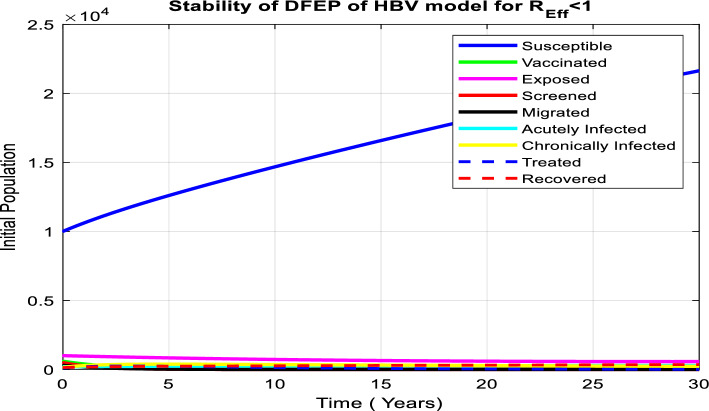
Graph of HBV model for ${\mathrm{R}}_{\mathrm{Eff}}<1.$

It is known that reproduction numbers are used to measure the transmission potential of a disease. From Fig. 4, it is seen that in 30 years, the number of susceptible populations increases, whereas the number of the remaining classes decreases to zero. Effective intervention strategies such as vaccination, screening, and treatment of HBV help to make the disease non-endemic in society by making effective reproduction numbers less than a unity. This shows that by making effective reproduction numbers less than unity by interpreting the intervention strategies we discussed above, we can control the transmission of HBV in society. The biological meaning of this simulation is that when effective reproduction number is less than one, then the number of infected individuals decreases where as the number of susceptible individuals increases.

**Figure 5:**
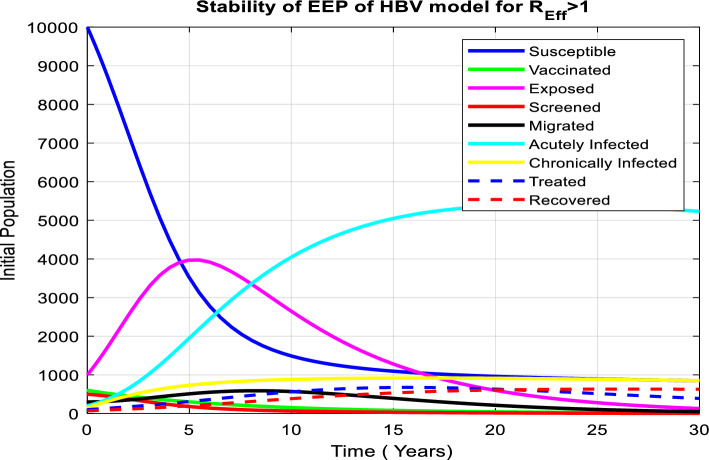
Stability of EEP of HBV model for ${\mathrm{R}}_{\mathrm{Eff}}>1.$

As illustrated in Fig. 5 above, the infectious classes, acutely infected class and chronically infected class increase, whereas the remaining classes decrease when the effective reproduction number is greater than unity. This shows that when a single infectious individual infects an averagely greater number of susceptible individuals in his or her lifetime, the number of infected and infectious individuals increases. The figure above shows us that the EEP of HBV is globally asymptotically stable because all the compartments except acutely and chronically infected classes converge to zero as time *t* goes to infinity. The biological meaning of this simulation is that when effective reproduction number is greater than one then the number of carriers and infectious individuals increases and that of the rest decreases.

## Result and conclusion

In this work, we considered a mathematical model consisting of nine compartments, namely: susceptible, vaccinated, exposed, screened, migrated, acutely infected, chronically infected, treated, and recovered classes. We calculated both disease-free and endemic equilibrium points of the model and proved their local and global stability. By using the next-generation matrix, we calculated the effective reproduction number. In the sensitivity analysis part, we found the perinatal infection rate, vaccination rate, contact rate, treatment rate, and screening rate to be the most influential parameters in changing the value of the effective reproduction number and changing the endemicity of the disease. We have also shown stability at both disease-free and endemic equilibrium points. We discussed the significance of subsequent vaccination of susceptible classes in order to fight against the transmission of HBV. We have shown the contribution of screening of migrants in decreasing the number of acutely infected classes. The result infers that vertical transmission has a role in suppressing the number of chronic infections. It is also shown that the effective reproduction number of HBV is significantly decreased by applying effective intervention strategies such as vaccination, treatment, and screening.

It also shows that for no intervention, ${R}_{Eff}=4.88$ and when the three intervention strategies are applied, ${R}_{Eff}=0.94$ showing the efficiency of those intervention strategies in reducing transmission of HBV. It is shown that by keeping perinatal infection rate $\gamma <0.308$, it is possible to control transmission of the virus. The result also shows significance of treatment in decrement of effective reproduction number. Maximizing $\eta >0.583$, it is possible to make ${R}_{Eff}<1.$ Essence of screening of exposed class in controlling effective reproduction number is also discussed. Effect of vaccinating the entire population to making effective reproduction number less than a unity is also shown. By keeping vaccination rate above 0.463 can enable to reduce transmission of HBV. The other contributing factor in increasing of effective reproduction number of HBV is contact rate. Graphically, the result has shown us that keeping contact rate below *0.49* enables to keep effective reproduction less than one.

The most important approach for preventing HBV infection is the use of the hepatitis B vaccine. The World Health Organization recommends frameworks such as raising awareness, promoting partnerships, and mobilizing resources based on evidence-based policy and data for action. The stakeholders should initiate and provide society with effective screening assays for surveillance, vaccines for prevention, and antiviral drugs that significantly improve patient clinical outcomes. The success and high coverage of universal HBV vaccination and the aging cohorts of patients with chronic hepatitis B will result in reductions in the incidence and prevalence of chronic hepatitis, cirrhosis, and probably hepatocellular carcinoma. This can be hastened by the excellent progress in treatment results for chronic hepatitis B patients. Screening migrants from countries with high prevalence rates to countries with low prevalence rates of HBV infection is also a critical intervention to prevent HBV transmission to the population of the hosting nations. Transmission prevention, screening, care, and treatment. This work contributes a lot on understanding and implementation of combined effect of vaccination, treatment and screening at different stages of HBV to combat transmission of the virus in the community and world at large. In general, we discovered that vaccination, screening of both exposed and migrating classes, and treating both chronically infected and screened classes are the best and most successful HBV control and suppression techniques. We can predict that implementing successful HBV vaccination, treatment, and screening initiatives will prevent new infections, ensure people have access to clinical care, and so lower the burden of infection at the individual, country, and regional levels. These advancements will help alleviate the tremendous social and economic burden of hepatitis B.

### Limitation of this work

The limitation of this work is that we didn’t consider reactivation of HBV after chemotherapy and liver transplantation. Other scholars can assume this case to extend this work.

## Data Availability

The data that support the findings of this study are available from W.J. John Edmunds. E-mail ID: https://www.sciencedirect.com/author/7006629172/william-john-edmunds”, Muhammad Altaf Khan. E-mail ID: https://pubmed.ncbi.nlm.nih.gov/?term=Khan%20MA%5BAuthor%5D”, Brian J McMahon, E-mail ID: https://pubmed.ncbi.nlm.nih.gov/?term=McMahon+BJ&cauthor_id=24266913. But, restrictions apply to the availability of these data, which were used under license for the current study, and so are not publicly available. Data are however available from the authors upon reasonable request and with permission of authors. Anyone interested in the data can contact the corresponding author.
